# Immunogenicity and protectivity of intranasally delivered vector-based heterologous prime-boost COVID-19 vaccine Sputnik V in mice and non-human primates

**DOI:** 10.1080/22221751.2022.2119169

**Published:** 2022-09-26

**Authors:** Amir I. Tukhvatulin, Ilya V. Gordeychuk, Inna V. Dolzhikova, Alina S. Dzharullaeva, Marina E. Krasina, Ekaterina O. Bayurova, Daria M. Grousova, Anna V. Kovyrshina, Alla S. Kondrashova, Daria V. Avdoshina, Stanislav A. Gulyaev, Tatiana V. Gulyaeva, Andrey V. Moroz, Viktoria V. Illarionova, Ilya D. Zorkov, Anna A. Iliukhina, Artem Y. Shelkov, Andrei G. Botikov, Alina S. Erokhova, Dmitry V. Shcheblyakov, Ilias B. Esmagambetov, Olga V. Zubkova, Elisaveta A. Tokarskaya, Daria M. Savina, Yulia R. Vereveyko, Anastasiya S. Ungur, Boris S. Naroditsky, Aydar A. Ishmukhametov, Denis Y. Logunov, Alexander L. Gintsburg

**Affiliations:** a Federal State Budget Institution “National Research Centre for Epidemiology and Microbiology named after Honorary Academician N F Gamaleya” of the Ministry of Health of the Russian Federation, Moscow, Russia; b Chumakov Federal Scientific Center for Research and Development of Immune-and-Biological Products of Russian Academy of Sciences, Moscow, Russia; c Sechenov First Moscow State Medical University, Moscow, Russia

**Keywords:** Sputnik V, Gam-COVID-Vac, intranasal vaccine, SARS-CoV-2, COVID-19

## Abstract

Although unprecedented efforts aiming to stop the COVID-19 pandemic have been made over the past two years, SARSCoV-2 virus still continues to cause intolerable health and economical losses. Vaccines are considered the most effective way to prevent infectious diseases, which has been reaffirmed for COVID-19. However, in the context of the continuing virus spread because of insufficient vaccination coverage and emergence of new variants of concern, there is a high demand for vaccination strategy amendment. The ability to elicit protective immunity at the entry gates of infection provided by mucosal vaccination is key to block virus infection and transmission. Therefore, these mucosal vaccines are believed to be a “silver bullet” that could bring the pandemic to an end. Here, we demonstrate that the intranasally delivered Gam-COVID-Vac (Sputnik V) vaccine induced a robust (no less than 180 days) systemic and local immune response in mice. High immunogenic properties of the vaccine were verified in non-human primates (common marmosets) by marked IgG and neutralizing antibody (NtAb) production in blood serum, antigen-specific Tcell proliferation and cytokine release of peripheral blood mononuclear cells accompanied by formation of IgA antibodies in the nasal mucosa. We also demonstrate that Sputnik V vaccine can provide sterilizing immunity in K18-hACE2 transgenic mice exposed to experimental lethal SARS-CoV-2 infection protecting them against severe lung immunopathology and mortality. We believe that intranasal Sputnik V vaccine is a promising novel needle-free mucosal vaccine candidate for primary immunization as well as for revaccination and is worth further clinical investigation.

## Introduction

More than two years have passed since the novel coronavirus (SARS-CoV-2) infection was declared by the WHO a pandemic, but coronavirus disease 2019 (COVID-19) still poses a serious health threat around the globe [1]. The initial goal of achieving population immunity, which is supposed to end the COVID-19 pandemic, requires substantial efforts made in two main directions: (i) by reaching sterilizing immunity in the vaccine-willing group (which has mostly already been immunized) and (ii) attracting non-immune vaccine-hesitant individuals into the vaccination campaign. Therefore, developing the next generation of COVID-19 vaccines addressing these aims is urgently needed.

Considering that SARS-CoV-2 invades through the upper respiratory tract epithelium intranasal (IN) COVID-19 vaccines specifically targeting the entry gates of infection by inducing robust local mucosal immunity might be superior to intramuscular (IM) vaccines [2].

Suppression of viral infection and subsequent replication in the upper airway mucosa, which is attributed to intranasal vaccines [3], is key to meeting several ultimate goals of vaccination, such as preventing the disease onset, limiting virus transmission (ideally reducing R0 to the desired value <1), as well as preventing virus evolution. IN vaccines also possess several no less important socio-economic benefits that include higher patient compliance (being especially important for young children), lower costs as there is no need to employ trained medical personnel as well as to ensure sterile conditions during vaccination, thus facilitating mass immunization in low-income countries. Slow vaccination pace with the great lag (as of 6 June 2022, only 22% of the population has received at least one dose of the vaccine) in Africa may be a stumbling stone for the ambitious strategy announced by the WHO to vaccinate 70% of the world’s population against COVID-19 by mid-2022 [4,5]. Thus, in a view of all the aforementioned advantages, there is a great demand for developing mucosal vaccines against COVID-19.

The two-component Gam-COVID-Vac (Sputnik V) vaccine based on recombinant human adenovirus type 26 and type 5 vectors, both carrying the S gene for SARS-CoV-2 spike glycoprotein (rAd26-S and rAd5-S), was launched in Russia in April 2020. Prime-boost intramuscular immunization with Sputnik V has proved to be safe, well-tolerated and highly effective against symptomatic (91.6%) COVID-19, and completely protective (100%) against severe cases [6,7]. Considering that both Ad26 and Ad5 vectors exhibit a tropism to the human respiratory tract, we hypothesized that Sputnik V vaccine might be highly effective when delivered intranasally. Here we describe the immunogenicity and protectivity of Sputnik V vaccine after prime-boost intranasal delivery compared to the conventional intramuscular administration using K18-hACE2 transgenic mice. Additionally, we further characterized Sputnik V immunogenicity and certain safety issues upon IN administration in non-human primates (Common marmosets).

## Results

### Differences in short-term humoral responses between the intranasally and intramuscularly administered Sputnik V vaccine in mice

**Figure 1.**
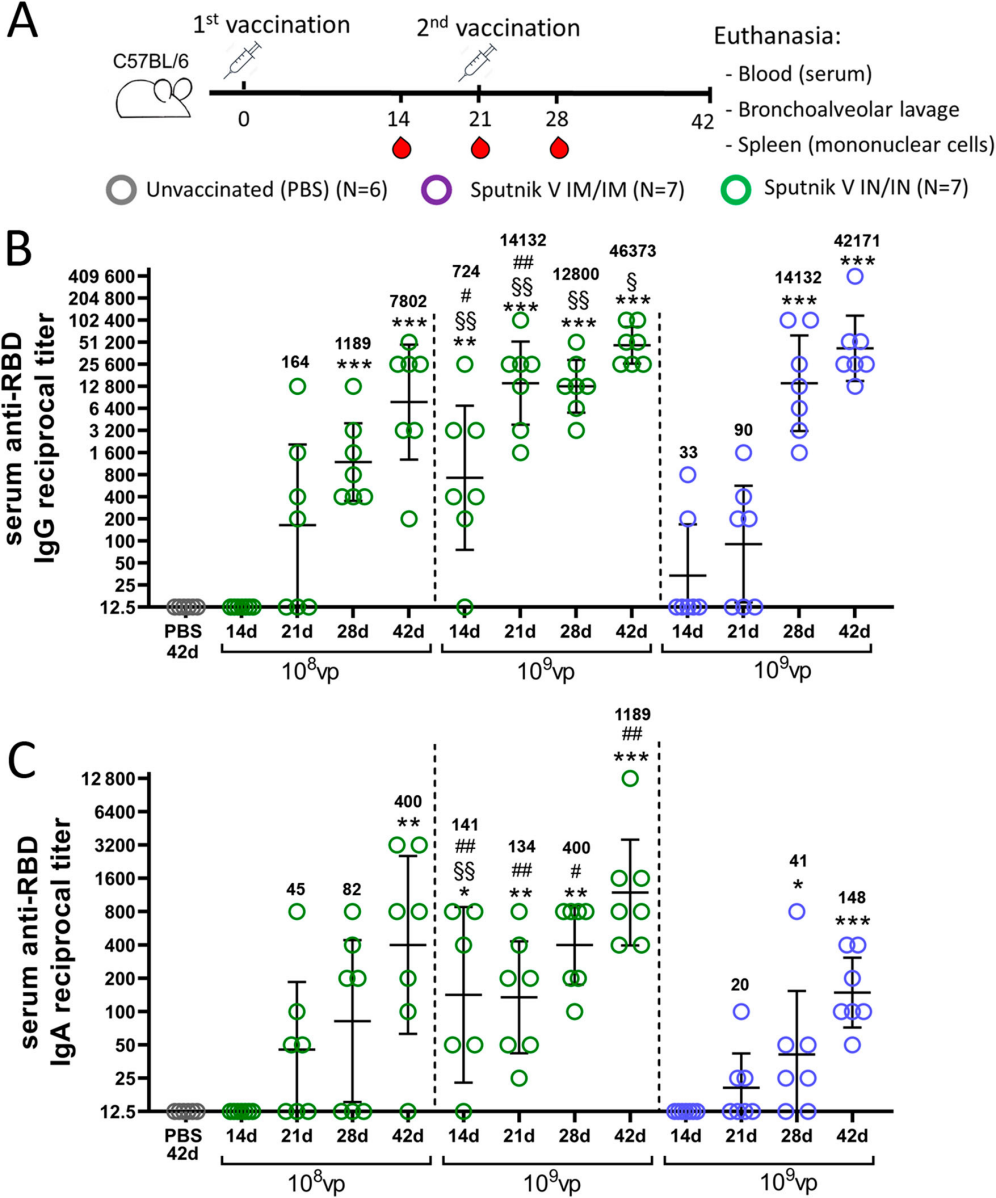
The kinetics of serum IgG and IgA responses after Sputnik V administration via intramuscular (IM) or intranasal (IN) route in C57BL/6 mice. (A) Study design. Mice (number of animals for each group is indicated in the legend) received prime-boost IM or IN vaccination with a 3-week interval. Non-vaccinated mice were injected with PBS. Mice were bled on days 14, 21 and 28 after the first immunization. On day 42 mice were sacrificed, blood and bronchoalveolar lavage (BAL) samples were collected to assess the humoral immune response. Splenic mononuclear cells were harvested to evaluate cell-mediated immune responses. The kinetics of RBD-specific IgG (B) and IgA (C) reciprocal titres in serum were detected at indicated time points by ELISA. Vaccine doses are shown below the X-axis. Dots represent individual data points. Horizontal lines represent geometric mean titres, whiskers are 95% CIs. Geometric mean titres are indicated above each data sample. No antigen-specific antibodies were registered in PBS-treated animals. Only the data on 42 d are shown for brevity. Significant differences between vaccinated and non-vaccinated animals were calculated using the Mann-Whitney U test and indicated with asterisks (* *p* < 0.05, ** *p* < 0.01, *** *p* < 0.005). Significant differences between different doses used for IN vaccination were calculated using the Mann-Whitney U test and indicated by section sign (§ *p* < 0.05, §§ *p* < 0.01). Significant differences between same doses used for IM and IN vaccination were calculated using the Mann-Whitney test and indicated with a hash (# *p* < 0.05, ## *p* < 0.01).

**Figure 2.**
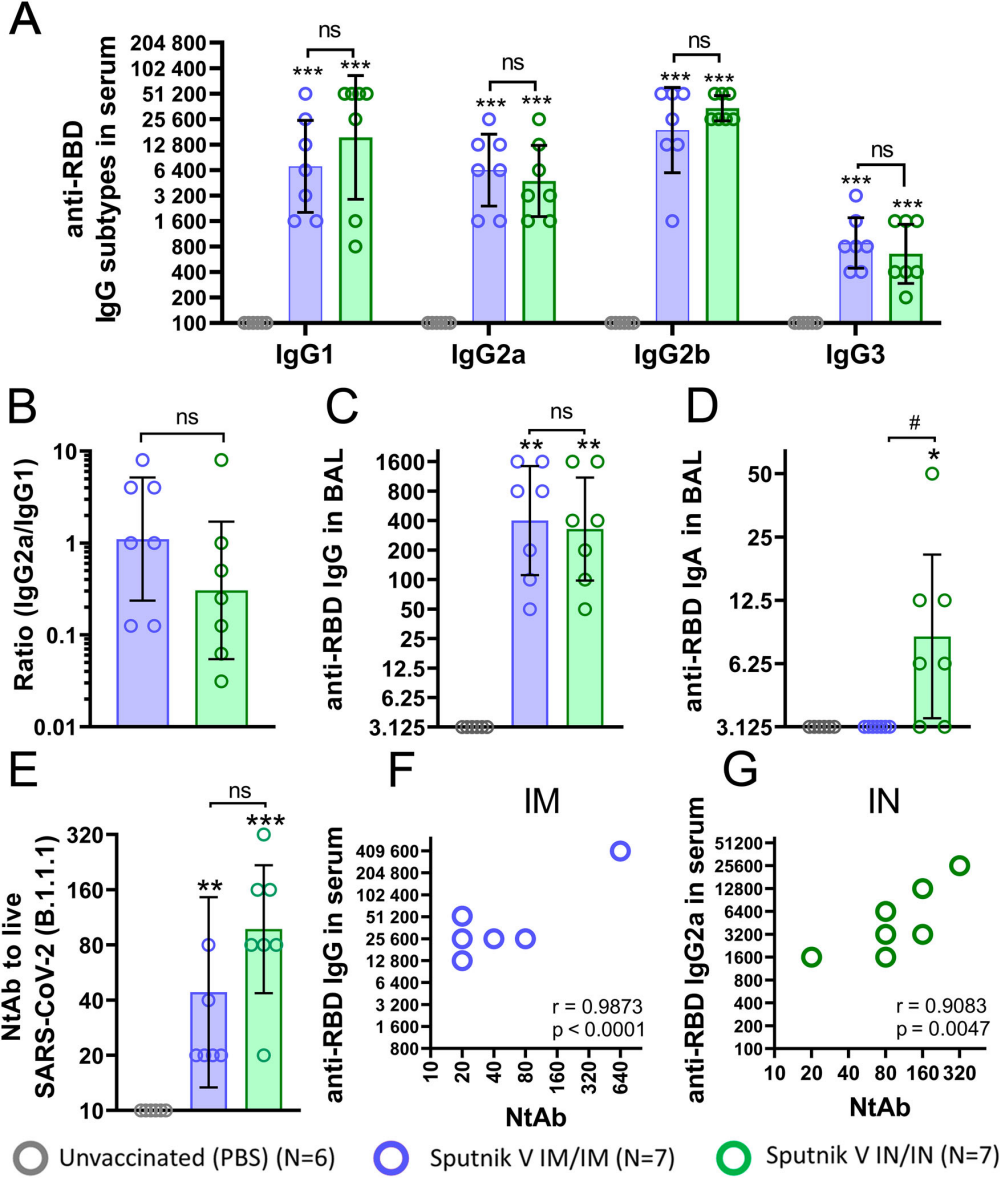
Characterization of the humoral immune response at endpoint in C57BL/6 mice that received Sputnik V vaccine via the intramuscular (IM) or intranasal (IN) route. The reciprocal titres of RBD-specific (A) IgG subtypes and the (B) IgG2a/IgG1 ratio detected in serum by ELISA. The reciprocal titres of RBD-specific (C) IgG or (D) IgA antibodies detected in BAL by ELISA. (E) The reciprocal neutralizing antibody (NtAb) titres (IC50) in the mouse sera. The correlation between (F) total IgG and NtAb titres in serum of IM vaccinated mice and (G) IgG2a and NtAb titres in serum of IN vaccinated mice was determined. Bars represent the geometric mean value for each group with 95%CI. Dots show individual data points. Significant differences between vaccinated and non-vaccinated animals were calculated using the Mann-Whitney U test and indicated with asterisks (* *p* < 0.05, ** *p* < 0.01, *** *p* < 0.005). # (*p* < 0.05) indicates significant difference between IM or IN vaccinated animals (Mann-Whitney U test). The Spearman rank correlation coefficient (r) and the *p* value between RBD-binding and virus-neutralizing antibodies are shown.

First, we studied the temporal systemic humoral response in mice after IN immunization compared to the original IM route of immunization with Sputnik V vaccine. For IN immunization, we used the same dose (10^9^vp) as that for IM as proposed for human trials, as well as a tenfold lower dose (10^8^ vp) to detect dose-dependent differences. Titres of SARS-CoV-2 anti-spike (S) protein receptor binding domain (RBD) IgG and IgA antibodies were assessed in limited volumes of blood collected on days 14, 21 and 28 from live anaesthetized mice, as well as on day 42 when the mice were sacrificed and detailed analysis of the humoral and cell-mediated responses was performed (Figure 1(A)). By day 42, the same vaccine doses (10^9^vp) administered via the IM or IN route elicited comparable serum reciprocal titres of IgG (the Geometric Mean Reciprocal Titer (GMRT) being 42,171 and 46,373, respectively, *p* = 0.75), but not IgA (GMRT 148 and 1189, respectively, *p* < 0.005) and the GMRTs of both antibody types were rising throughout the observation period (Figure 1(B, C)). Interestingly, IN administration of a tenfold lower dose resulted in significantly lower IgG titres at the same time point (GMRT 7802, *p* = 0.09), while still inducing higher titres of IgA (GMRT 400), compared to IM immunization with a higher dose (GMRT 148, *p* = 0.17). Simultaneously, the kinetics of antibody levels differed substantially over time between the immunization routes. IN vaccination with 10^9^vp significantly increased the antigen-specific IgG and IgA levels in mouse serum starting from day 14 after the first immunization, contrary to IM vaccination where the humoral immune response was indistinguishable from that in non-vaccinated mice up to day 21 and then was significantly boosted by the second immunization by days 28 and 42. A detailed analysis of the humoral response on day 42 showed that the characteristics of antigen-specific IgG response including titres of IgG subtypes, the ratio between IgG2a and IgG1 subtypes (associated with Th1 or Th2 polarization, respectively) as well as IgG titres in bronchoalveolar lavage (BAL) (Figure 2(A–C)), were comparable for the IM and IN routes. The only but fundamental difference between the two routes of immunization was observed in the lung mucosal response, where only IN vaccination resulted in significant elevation of RBD-specific secretory IgA in BAL (Figure 2(D)). These data show that both IM and IN vaccination can induce comparable levels of systemic and lung mucosal IgG response, but IgA antibodies (in serum, and especially in lung mucosa) could be effectively generated only after IN inoculation.

One of the main antibody functions in complete antiviral protection is virus neutralization. We noticed that the differences in serum IgA titres coincided with a twofold (but not statistically significant) increase in serum virus-neutralizing activity for IN vaccination compared to vaccination through the IM route (GMRT 97 and 44, respectively) (Figure 2(E)). These data are in line with the well-known finding that IgA antibodies provide virus neutralization along with the IgM and IgG classes [8].

The level of antigen-specific IgG antibodies is also a good marker for predicting the presence of neutralizing antibodies (NtAbs). The observed correlation makes rational ground for using various IgG ELISA tests in clinics to determine the overall individual protection level that in its turn is strongly bound to the NtAb titre [9]. However, we have found that this observation is valid only for IM vaccination (Figure 2(F)). Neither IgG nor IgA titres in serum or BAL correlated with NtAb titres upon IN vaccination (Supplementary Tables S1-2). With this background it was surprising to detect a strong correlation (*p* = 0.0047, *r* = 0.9083) between the IgG2a subtype and NtAb titres (Figure 2(G)). This brings us to a conclusion that anti-RBD IgG2a antibodies might be a reliable surrogate marker for assessing serum neutralizing activity in humans upon IN vaccination.

### Intranasal administration of Sputnik V establishes long-term humoral immunity in mice

**Figure 3.**
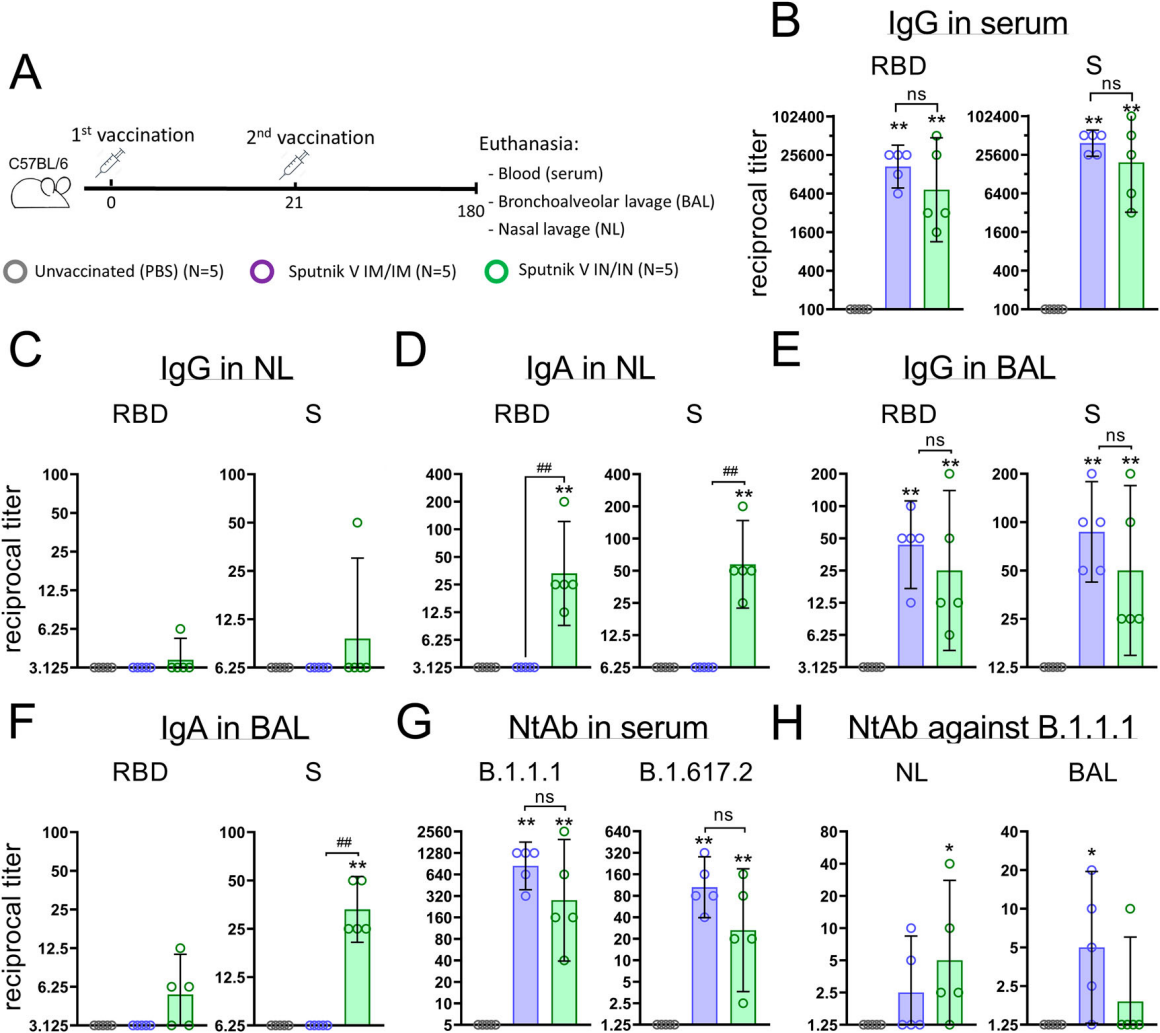
Humoral immune response of C57BL/6 mice 180 days after intramuscular (IM) or intranasal (IN) vaccination with Sputnik V. (A) Study design. Mice (the number of animals for each group is indicated in the legend) received prime-boost IM or IN vaccination with a 3-week interval. Non-vaccinated mice were injected with PBS. On day 180, mice were sacrificed; blood, nasal and bronchoalveolar lavages were collected to assess the humoral immune response. (B) The reciprocal anti-RBD and anti-S IgG titres in blood serum. The reciprocal anti-RBD and anti-S IgG (C) and IgA (D) titres in nasal lavages (NL) and in bronchoalveolar lavages (BAL) (E-F). (G) The reciprocal neutralizing antibody (NAb) titres (IC50) against live B.1.1.1 and B.1.617.2 SARS-CoV-2 variants in the mouse serum. (H) The reciprocal NAb titres (IC50) against live B.1.1.1 SARS-CoV-2 variant in NL and BAL samples. Bars represent the geometric mean for each group with 95%CI. Dots show individual data points. Significant differences between vaccinated and non-vaccinated animals were calculated using the two-tailed Mann-Whitney U test (*p < 0.05, ** *p* < 0.01). Hashes indicate significant differences between IN and IM vaccinated animals (## *p* < 0.01, Mann-Whitney U test).

To estimate the durability of systemic and local humoral immune response in mice, we collected blood as well as nasal and bronchoalveolar lavages on day 180 after first IM or IN immunization using the same dose (10^9^vp) of vaccine (Figure 3(A)). Compared to day 42, the decrease in anti-RBD IgG reciprocal GMRTs was less pronounced in the IM group (from 42,171 to 19,200), than in the IN group (from 46,373 to 16,960), but remained statistically insignificant between different vaccination routes (Figure 3(B)).

The mucosal immune response in the upper respiratory tract was detected only after IN vaccination and was presented by IgA antibodies (Figure 3(C,D)). In the lower respiratory tract, comparable significant IgG levels were maintained after both immunization routes (Figure 3(E)), but IgA levels were again registered only after IN vaccination (Figure 3(F)). Interestingly, IgA reciprocal GMRTs in NL (75) were higher than those in BAL (35) after IN vaccination, although the differences were not statistically significant.

Evaluation of virus neutralizing capacity for the serum samples showed an increase in NtAb GMRTs from day 42 to day 180 for both IN (128 and 712, respectively) and IM (120 and 960, respectively) vaccination routes (Figure 3(G)). IN vaccination resulted in statistically insignificant (but slightly lower) NtAb titres against ancestral B.1.1.1 and B.1.617.2 (Delta variant) strains compared to IM vaccination. Evaluation of virus neutralizing activity at mucosal sites revealed that statistically distinct NtAb titres were registered in the upper respiratory tract of IN vaccinated animals (GMRT 11.3) and in the lower respiratory tract in IM vaccinated animals (GMRT 7.8) (Figure 3(H)). As only IgA antibodies were detected in NL after IN vaccination, these results support the finding that sole IgA antibodies could neutralize the SARS-CoV-2 virus providing effective immune protection [10]. On the other hand, in the absence of IgA antibodies, the neutralizing activity in the lower respiratory tract after IM vaccination is defined by IgG. This conclusion is in line with the previously published papers delegating the dominant role in viral protection in the lower respiratory tract to IgG [11].

Thus, both IN and IM vaccination establish a long-term systemic humoral immunity in mice with marked differences of immune response in the upper and lower airway mucosa.

### Intranasally administered Sputnik V induces marked systemic and local cell-mediated immune response in mice

**Figure 4.**
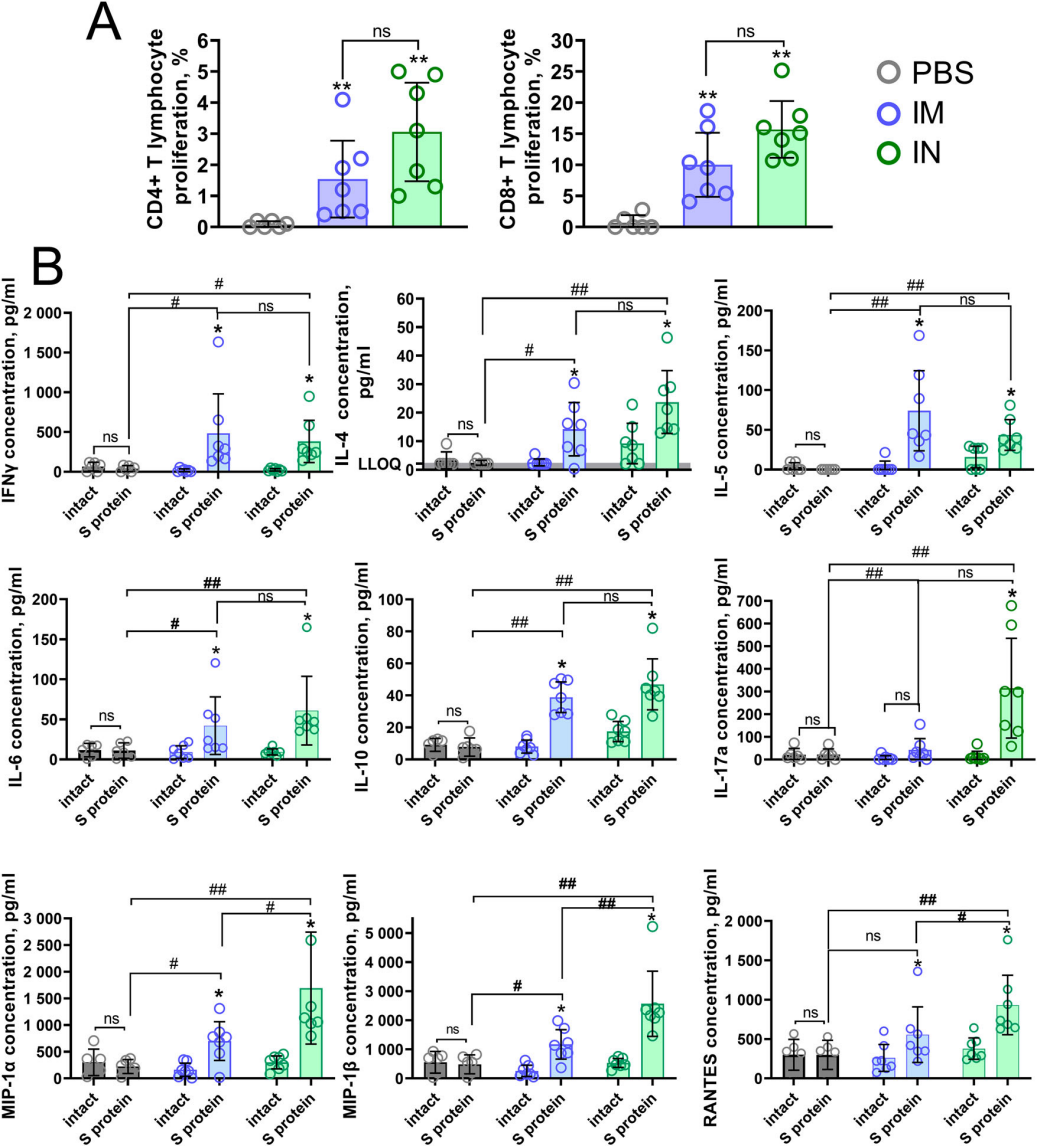
Antigen-stimulated CD4 + and CD8 + T cell proliferation and cytokine production in splenocytes from С57BL/6 mice that received Sputnik V via the intramuscular (IM) or intranasal (IN) route. Placebo mice were injected twice with PBS. (A) CD4 + and CD8 + T cell proliferation was calculated as the difference (Δ) in % of proliferating (CFSE dim) lymphocytes between stimulated vs non-stimulated cells for each animal. (B) Graphs show the absolute cytokine levels in pg/mL. Dots show individual data points. Each bar represents the mean value per group ± SD (error bars). *P*-values in cytokine response between stimulated and non-stimulated cells within one group were calculated using the Wilcoxon signed-rank test (* *p* < 0.05). Significant differences between animals from groups were calculated using the Mann-Whitney U test (# *p* < 0.05, ## *p* < 0.01). The lower limit of quantification (LLOQ) is indicated with a grey line.

The cell-mediated adaptive immunity represents a critical complementary to the humoral response or, in some cases, even the sole (for inborn immunodeficiencies) measure providing immune protection against COVID-19. Whereas many questions are still under thorough investigation (including what characteristics of antigen-specific T cells could be indicative for clinical outcome), it is considered that both CD4+ and CD8+ T cells contribute to protective immune responses against SARS-CoV-2 [12]. Systemic cell-mediated immunity elicited by IM and IN vaccination was characterized by measuring CD4+ and CD8+ T cell proliferative responses, as well as concentration of cytokines and chemokines after antigen restimulation of murine splenocytes *in vitro*. We found that irrespective of immunization route, Sputnik V elicits a strong T cell proliferative response where CD8+ T cells predominate over CD4+ T cells (Figure 4(A)). IN vaccination elicited a higher (but statistically insignificant) CD4+ T cell response (3.1% and 1.5%, respectively) as well as CD8+ T cell response (15.7% and 10.0%, respectively) than IM vaccination. Such CD8+ biased T cell response specific for all adenoviral-based vaccines [13] was shown to be important in protecting against the development of severe COVID-19 [14,15].

In parallel, concentrations of 23 cytokines were measured in conditioned culture medium upon antigen restimulation of cells (Figure 4(B)). We detected that secretion of both Th1 (IFNɣ) and Th2 cytokines (IL-4, IL-5, IL-6, and IL-10), were elevated in mice that received IM or IN vaccination. Distinctively increased production of IL-17A (Th17) was observed only in mice that received IN vaccination, implying the formation of mucosal immune response after IN delivery of Sputnik V. The levels of chemokines such as MIP-1α (CCL3), MIP-1β (CCL4) and RANTES (CCL5) were upregulated in all the vaccinated groups, but IN vaccination resulted in their significantly higher production compared to IM. As there is a great concern about the emergence of cytokine storm syndrome during COVID-19 infection, the observed data have great importance. According to the collected and reviewed data, the levels of all three chemokines negatively correlate with disease severity and are likely to be associated with antigen-specific T cell formation, recovery and resolution of inflammation [16].

**Figure 5.**
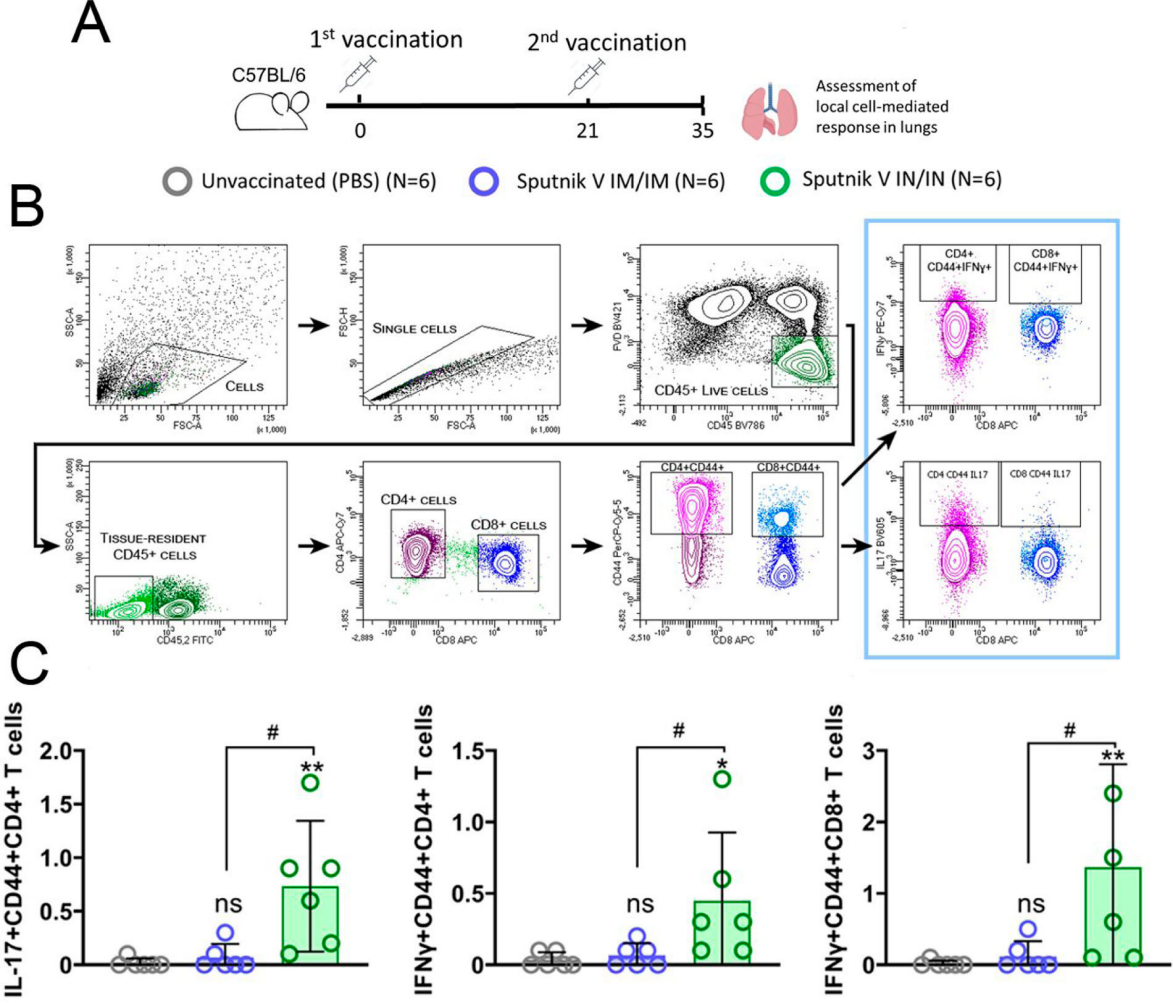
Flow cytometry analysis of tissue-resident lung lymphocytes from С57BL/6 mice that received intramuscular (IM) or intranasal (IN) Sputnik V vaccine. (A) Study design. Mice (the number of animals for each group is indicated in the legend) received prime-boost IM or IN vaccination with 3-week interval. Non-vaccinated mice were injected with PBS. On day 35 mice were sacrificed for evaluating the local immune response in lung parenchyma. (B) Gating strategy for assessing IL17 + and IFNɣ+ CD4 and CD8 tissue-resident lymphocytes. Lymphocytes were gated using the forward and side scatter characteristics. Live CD45 + cells were then obtained from the single cells gate. Intravenously administered anti-CD45-FITC antibodies were used for distinguishing tissue-resident (CD45.2-) and intravascular (CD45.2+) live lymphocytes. IL17 and IFNɣ expression was detected in the CD44 + fraction of CD4 + and CD8 + lymphocytes. (C) The individual percentages of IL-17+, IFNy+ CD4 + CD44 + and IFNy+ CD8 lymphocytes are shown by dots. Each bar represents the mean value per group ± 95%CI (error bars). Significant differences between vaccinated and unvaccinated animals were calculated using the Mann-Whitney test and indicated with asterisks (* *p* < 0.05, ** *p* < 0.01). # (*p* < 0.05) indicates significant difference between IM or IN vaccinated animals (Mann-Whitney test).

Next, we assessed the induction of cell-mediated immune responses in the lungs by the different vaccination routes (Figure 5(A)). Intravital intravascular staining with anti-CD45-FITC antibodies was used to differentiate between blood-borne and resident lymphocytes present in the lung tissue (Figure 5(B)) [17]. As Th1/Th17 responses are known to play important roles in the outcome of viral infections, we quantified IL-17+ and IFNɣ+ CD4+ and IFNɣ+ CD8+ lymphocytes in lung tissue after IN and IM vaccination. Similar to the IgA levels, IN vaccination resulted in significantly higher percentages of IL17+ (mean, 0.73%), IFNɣ+ (0.45%) CD4+ CD44+ and IFNɣ+ (1.37%) CD8+ CD44+ cells in the lung tissue compared to statistically undistinguishable levels in the IM group (Figure 5(C)). There were no differences between any studied groups in cytokine-positive cells without intravascular staining discrimination (data not shown).

Collectively, these findings demonstrate that in the mouse model, intranasal and intramuscular administration of Sputnik V vaccine elicits comparable magnitude of central antigen-specific immunity (expressed by NtAbs, cell-mediated response) while only intranasal vaccination forms humoral and cell-mediated response in lungs.

### Intranasal administration of Sputnik V is well-tolerated and induces both systemic and local humoral response in NHPs

**Figure 6.**
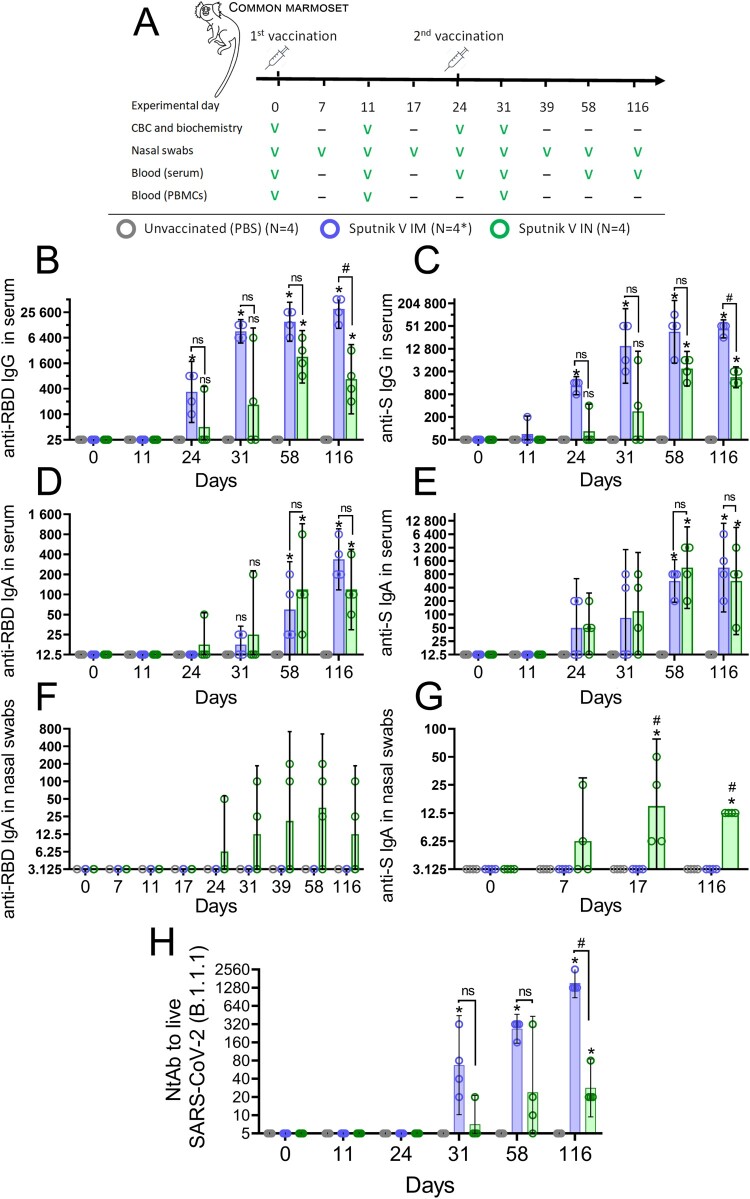
Humoral immune response of common marmosets that received Sputnik V via the intramuscular (IM) or intranasal (IN) route. (A) NHP study design. NHP were vaccinated twice with Sputnik V vaccine (2 × 10^10^vp) intramuscularly (IM) or intranasally (IN) with 24-day interval. The placebo group received PBS both IM and IN on the same days. Welfare monitoring was conducted throughout the study. Blood and nasal swabs were collected as indicated to assess cellular and humoral immune responses. * One animal (#0701) was excluded from cell mediated response analysis on days 11 and 31 because of blood clotting and low PBMC yield. The anti-receptor-binding domain (RBD) as well as anti-S IgG (B,C) and IgA (D,E) responses in the serum of marmosets prior and after the first and second IM and IN immunizations by ELISA. (F) Anti-receptor-binding domain (RBD) and (G) anti-S IgA responses in the nasal swabs of marmosets prior and after the first and second IM and IN immunizations. Bars represent geometric mean for each group with 95%CI. Dots show individual data points. Significant differences between vaccinated and non-vaccinated animals were calculated using the two-tailed Mann-Whitney U test (*p < 0.05). Hashes indicate significant differences between IN and IM vaccinated animals (# *p* < 0.05, Mann–Whitney test).

The differences in nasal anatomy and nasal antigen presentation between rodents and humans are well known [18,19]. Therefore, to support the conclusions obtained in rodent experiments we also evaluated the immunogenicity of intranasally administered viral vector vaccine using non-human primates (NHPs) whose structure of nasal mucosa is more relevant to that in humans. We used 12 adult common marmosets (*Callithrix jacchus*; CM) randomly assigned to three equal groups that: (i) received IM immunization, (ii) received IN immunization of the same dose (2 × 10^10^ vp, 1/5 of human dose) of Sputnik V vaccine; or (iii) received the same volume of placebo (PBS) both via the IM and IN route. Six days prior to vaccination, the baseline general health (rectal temperature, body weight, biochemical blood analysis, complete blood count (CBC)) and immunological (absence of pre-existing antibodies to SARS-CoV-2 virus as well as to Ad5 and Ad26 human adenoviruses) parameters were measured. Nasal swabs and blood samples were taken routinely at indicated time points to assess immunogenic as well as several safety parameters of Sputnik V administered via the IN and IM route (Figure 6(A)).

Throughout the study, we observed only two mild deviations and only between the IM and PBS injected groups (Supplementary Tables S3–S6). On Day 28 after the first immunization, a statistically significant (*p* = 0.013) decrease in body temperature (0.8°C) was observed in the animals from the IM group as compared to the placebo group. Furthermore, an increase of the percentage of lymphocytes in the CBC (*p* = 0.029) with a compensatory statistically significant decrease in the percentage of segmented neutrophils (*p* = 0.018) was observed in the IM group as compared to the placebo group on day 31 after the first immunization. However, both of these parameters lay within the normal range for CMs. These observations may indicate the development of the post-vaccinal immune response and correlate with side effects observed in clinical trials [20,21]. Importantly, there were no statistically significant differences in overall health condition, body weight and body temperature, biochemical parameters in serum and CBC between the animals that received IN Sputnik V vaccine compared to the placebo group. Therefore, the IN vaccination is exceptionally well-tolerated by non-human primates (*Callithrix jacchus*).

The magnitude of antigen-specific humoral response to IM and IN vaccination was evaluated by measuring antigen-specific IgG and IgA antibodies in marmoset blood as well as IgA antibodies in nasal swabs. As expected, the primate model showed the IgG kinetics after IM and IN vaccination that was different from the mouse model. Firstly, serum anti-RBD and anti-S IgG antibodies after IM vaccination were detected in animal at an earlier time point (GMRT 475 and 1400 on day 24, respectively) compared to the IN group (GMRT 118 and 137 on day 24, respectively) (Figure 6(B,C)).

Secondly, the magnitude of IgG response was generally higher at each time point in the IM group compared to the IN group, but differences became statistically significant only at the late time point (day 116). Between day 58 and day 116, anti-RBD and anti-S IgG titres continued to grow in the IM group (e.g. anti-RBD IgG GMRT increased from 17,600 to 35,200) whereas titres in the IN group the titres started to decrease after day 58 (anti-RBD IgG GMRT decreased from 3000 to 1150).

Contrary to IgG, the kinetics of blood titres of IgA were similar between the two experimental groups, reaching a statistically significant increase compared to the PBS group on day 58 and day 116 (Figure 6 (D,E)). Significant differences between the IN and IM groups were registered for IgA titres in nasal swabs. As observed in BAL of vaccinated mice, only IN vaccination resulted in formation of distinguishable levels of IgA antibodies in nasal swabs collected from CMs. Consistent with the notion that titres of S-specific antibodies are generally higher than those of RBD-specific antibodies, we registered statistically significant levels of anti-S IgA antibodies (but not anti-RBD IgA antibodies) in nasal swabs on days 17 and 116 (Figure 6(F, G)). It is noteworthy that robust anti-S IgA response in the upper airways was formed as early as on day 7 after the primary IN immunization, supporting the idea that mucosal vaccines are a good choice for urgent immune protection.

In accordance with antigen-binding antibody response in blood, IM vaccination induced formation of virus neutralization activity of sera earlier and at higher levels compared to IN vaccination. Thus, in the IN vaccinated group, a distinguishable level of NtAb was detected only on day 116 (GMRT 35), while IM vaccinated animals developed significantly higher NtAb levels (GMRT 1600) (Figure 6(H)). NtAb titres continued to grow in both IM and IN groups up to day 116, allowing us to infer that virus neutralization activity of the sera has not reached its maximum. We have not performed any correlation analysis between the titres of antigen-binding and virus-neutralizing antibodies because of the small animal sample.

### Intranasal administration of Sputnik V induces marked cell-mediated response in NHPs

**Figure 7.**
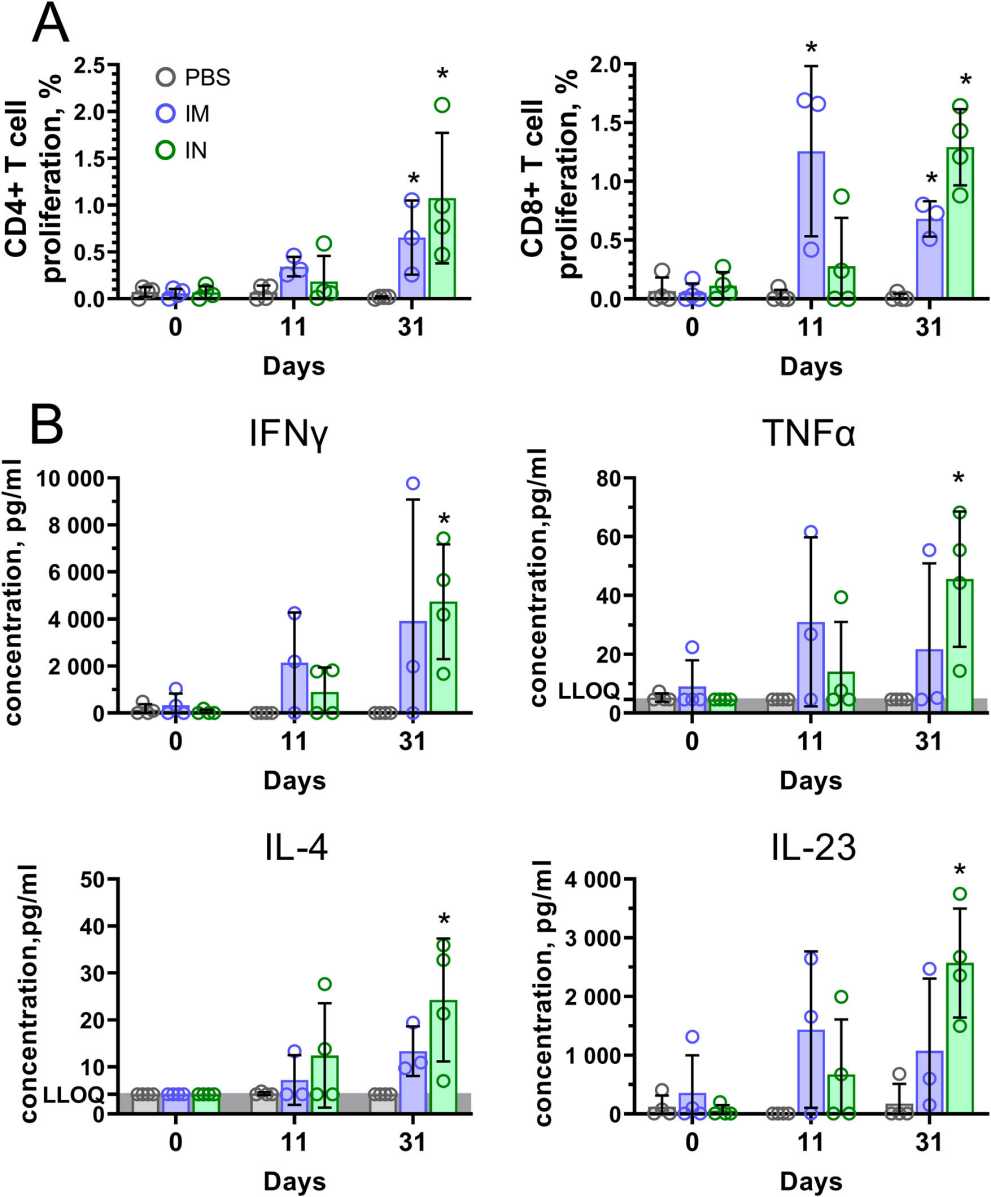
Antigen-stimulated CD4 + and CD8 + T cell proliferation and cytokine production in PBMCs of common marmosets that received Sputnik V via the intramuscular (IM) or intranasal (IN) route. (A) CD4 + and CD8 + T cell proliferation was calculated as the difference (Δ) in % of proliferating (CFSE dim) lymphocytes between stimulated vs non-stimulated cells for each animal. (B) The cytokine data were presented as the difference (delta) in cytokine concentrations between the samples with and without protein stimulation. Dots show individual data points. Each bar represents the mean value per group ± SD (error bars). Significant differences between vaccinated and non-vaccinated animals were calculated using the Mann-Whitney U test (* *p* < 0.05). NS, not significant.

Cell-mediated response measured by detection of T-cell proliferation and cytokine production by PBMCs after antigen restimulation was studied in marmosets before vaccination, 11 days after the first vaccination and 7 days after the second vaccination (31 days after the first vaccination). One animal from the IM group (ID #0701) was excluded from analysis because of blood clotting and, therefore, low PBMC yields. Excessive clotting is a typical observation for blood samples obtained from CMs [22]. Both IM and IN vaccination induced consistent formation of antigen-specific CD4+ (0.65% and 1.08%, respectively) and CD8+ (0.68% and 1.29%, respectively) T cells in peripheral blood on day 31 (Figure 7(A)). IN vaccination resulted in a higher T cell proliferative response compared to IM, however, the observed differences were not statistically significant. The functional activity of antigen-specific cells was characterized by expression of 14 cytokines. Consistent with the results obtained in mice, we observed significant production of Th1 (IFNɣ, TNFα), Th2 cytokines (IL-4), as well as Th17 (IL-23) cytokines by PBMCs of IN vaccinated animals (Figure 7(B)). Concentrations of other cytokines that are important to T cell- and B cell-mediated immunity but were elevated insignificantly upon antigen restimulation are presented in Supplementary Figure 1.

Having summarized the results of immunogenicity studies, we can say that IN vaccination with Sputnik V forms a strong systemic as well as local immune response in respiratory mucosa in both mice and non-human primates.

### Intranasal vaccination with Sputnik V reduces inflammation in the lungs of K18-hACE2 trangenic mice and establishes their resistance to lethal SARS-CoV-2 challenge

**Figure 8.**
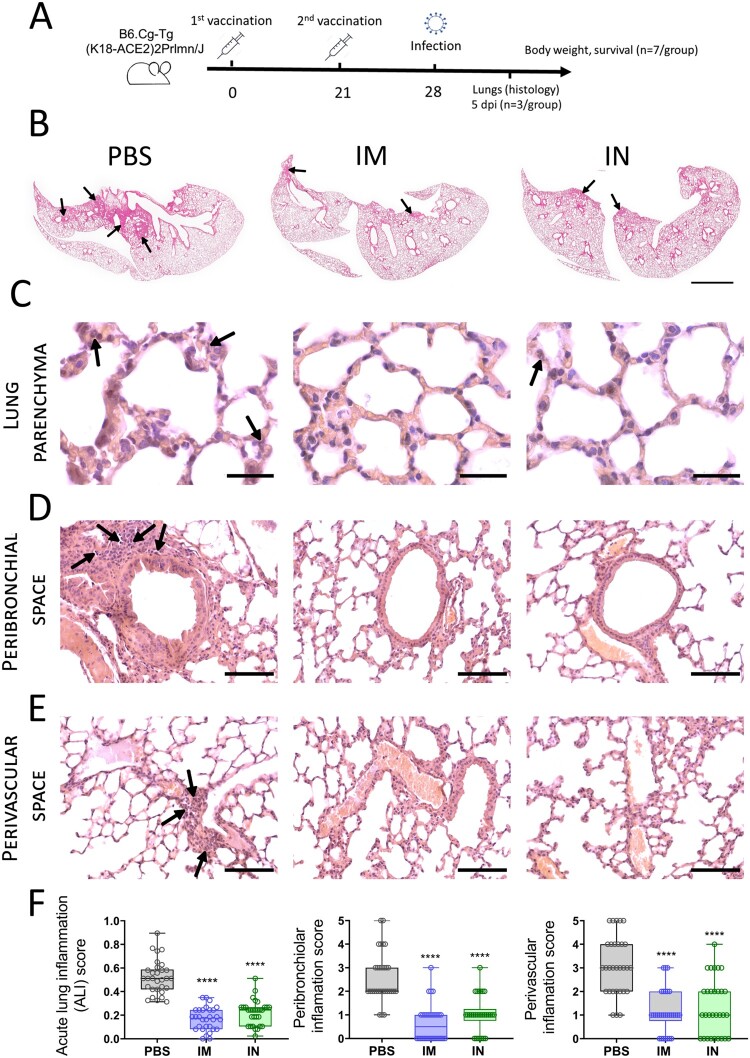
**S**ARS-CoV-2-mediated histopathological changes in the lungs of non-vaccinated, as well as IM or IN vaccinated K18-hACE2 mice. (A) Study design. (B). Haematoxylin and eosin (H&E) stained sections of mouse lungs, arrows pointing to inflammation foci; bar = 2 mm (C). Representative H&E sections of the lung parenchyma, arrows show neutrophils in the interalveolar septa; bar = 30 µm. Representative H&E sections aimed on (D) peribronchiolar and (E) perivascular spaces. Arrows indicate inflammatory infiltration. Bar = 100 µm. (F) Acute lung inflammation (ALI), peribronchiolar and perivascular scores from 10 random fields / 10 random bronchioles / 10 random vessels, respectively, for each of three mice in one experimental group (n = 30). Boxes show the interquartile range, whiskers show the range, and horizontal lines represent the median values. Dots show individual data points. Significant differences between vaccinated and non-vaccinated animals were calculated using by two-way ANOVA (**** *P* < 0.0001).

Having demonstrated high immunogenic properties of IN delivered Sputnik V vaccine, we finally studied the protective capacity of IN Sputnik V in the SARS-CoV-2 challenge model. K18-hACE2 mice (10 per group) received prime-boost IM or IN vaccination with a 3-week interval (Figure 8(A)). Mice from the placebo group were inoculated with PBS in the same volume. Mice were challenged intranasally with a lethal dose (10^5^ TCID_50_) of SARS-CoV-2 on day 28. The lungs from 3 mice were collected on day 5 post infection (5DPI) for performing histological analysis and evaluating the viral load.

**Figure 9.**
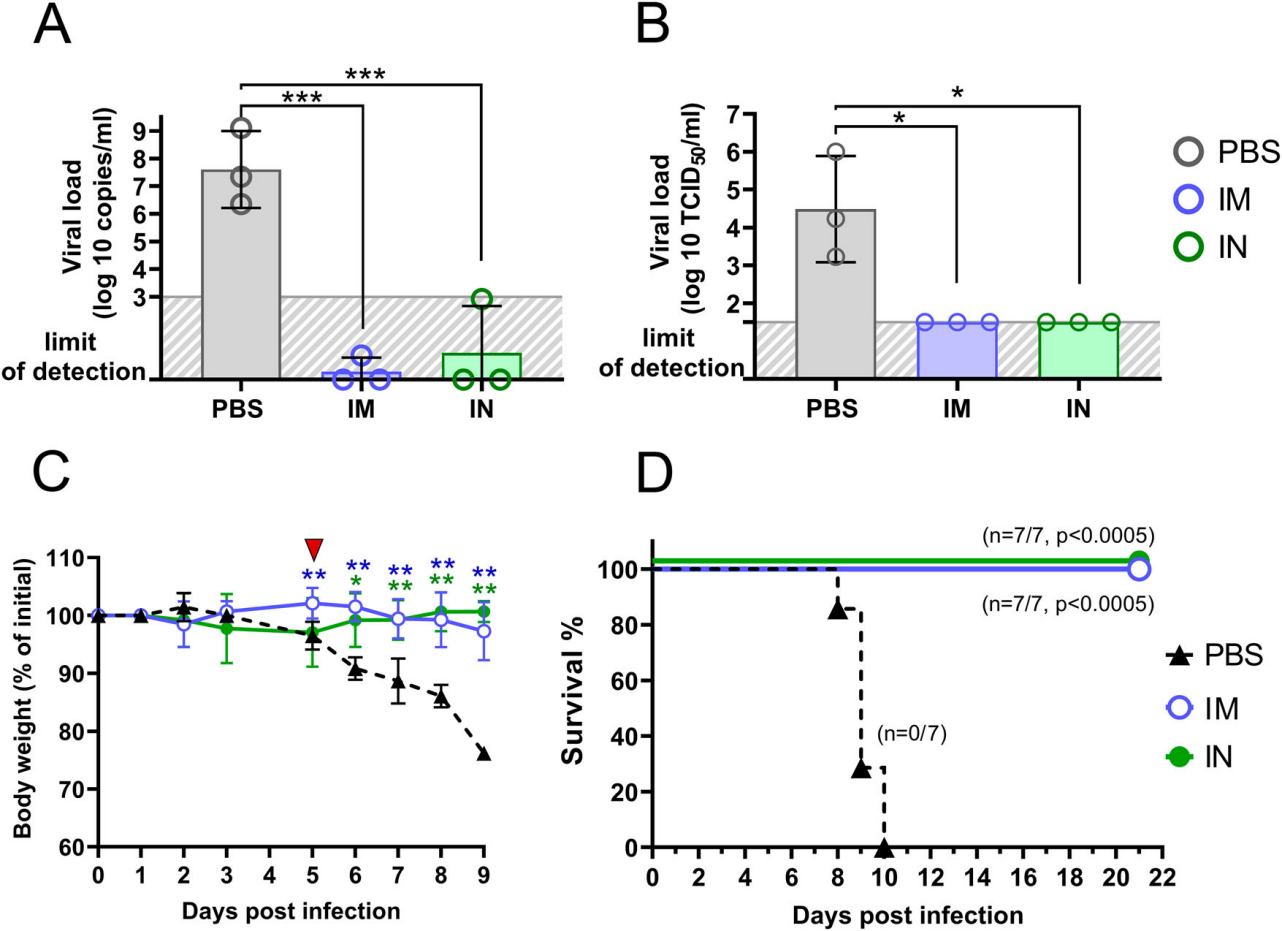
Vaccine protection against lethal SARS-CoV-2 infection in K18-hACE2 mice. Three mice per group were sacrificed and their lungs (right interior lobe) were collected at 5 dpi for analyzing of viral loads in homogenates determined by (A) genome equivalents using RT-qPCR and (B) virus titre using plaque assay on Vero E6 cells. Significant intergroup between groups were calculated using the Mann-Whitney test (* *P* < 0.05; *** *P* < 0.005). (C) percentage of initial body weight loss in mice. Significant differences between vaccinated and non-vaccinated animals were calculated by two-way ANOVA (* *P* < 0.05; ** *P* < 0.01). Red triangle depicts experimental endpoint for viral load and histological analysis. (D) The Kaplan-Meier survival curve of non-vaccinated mice and mice received IM or IN administration of Sputnik V vaccine. Significant differences in survival rates between vaccinated and non-vaccinated animals are indicated (Log-rank (Mantel–Cox) test).

We chose the 5DPI as a time point when virus load in lung tissue starts decreasing but still remains at a high level, while progression of pneumonia reaches its peak. It is also an important clinical bifurcation time point at which mice have marked signs of the disease (e.g. weight loss) but no deaths occur yet [23,24]. At whole slide images, a wide area of intense H&E staining was seen in lung sections of PBS-treated animals compared to a few small lesions in the IM and IN vaccinated groups (Figure 8(B)). More thorough histological analysis at higher magnification revealed that pathomorphological changes in the lung tissue mainly caused by massive inflammatory infiltration in alveolar septa as well as large-scale lesions of immune cells bordering blood vessels and bronchial tubes (Figure 8(C–E)). Having evaluated the grade of inflammation in lung parenchyma (the ALI score), and precisely peribronchial as well as perivascular areas using the corresponding scoring systems, we found that both IM and IN vaccination with Sputnik V significantly reduced the grade of immunopathology in all the areas (Figure 8(F)). We have not detected any significant difference between IM and IN vaccinated animals. Although residual immune infiltrate (e.g. solitary neutrophils in alveolar septa) remained at 5DPI in the lungs of vaccinated animals, viral loads measured by RT–PCR (expressed viral genome copies) (Figure 9(A)) or by cell culture technique (TCID_50_/ml) (Figure 9(B)) were at undetectable levels compared to the high levels in the PBS-treated group. We believe that the observed discrepancy was caused by the nature of the immune response that needs time to utilize negative feedback loops to maintain physiological homeostasis after complete pathogen clearance rather than by the high dose of virus (that was also used for challenge study) [25].

The remaining seven mice per group were daily monitored for survival and body weight upon lethal SARS-CoV-2 challenge. Mice in the placebo group showed steady weight loss starting from 5 DPI (Figure 9(C)). Since 6 DPI, there was a significant difference in weight between control non-vaccinated and both IM and IN vaccinated groups. Progressive weight loss was associated with rapid mortality at 8–10 DPI in the placebo group. None of vaccinated animals died or reached high disease scores requiring humane intervention (Figure 9(D)).

Taken together, intranasal administration of Sputnik V can establish sterilizing immunity, ensure protection against lung immunopathology, and fully prevent mortality upon experimental SARS-CoV-2 infection.

## Discussion

According to the updated landscape of COVID-19 candidate vaccines published by the WHO there are 156 different vaccines in ongoing clinical trials; most of those (137 or 88%) are parental (subcutaneous, intradermal and intramuscular) and only 13 vaccines (8%) are designed for mucosal (oral, aerosol, inhaled, intranasal) administration (other six vaccines with mixed or unspecified routes of administration were excluded from analysis) [26].

The reasons for such disproportion apparently originate from the intrinsic nature of the mucosal immune system. Being the first line of defense in the human body, mucosal surfaces act as a complex multilayered barrier that cannot be crossed even by highly invasive pathogens naturally selected for this purpose, not to mention vaccine compositions. Antigen delivery to antigen-presenting cells (APC) for the purpose of vaccination is significantly hampered by the thick and mobile mucus layer, enzymatically active environment as well as simple dilution on its way. Even if such an efficient barrier was penetrated, the mucosal immune system operates with a high threshold (known as the mucosal immune tolerance, which is also observed for nasal mucosa) that distinguishes between a pathogenic infection that is worth to respond to and a basal harmless antigenic stimulus. Thus, for efficient APC activation, it is highly desirable that characteristics of vaccine antigen (e.g. its nature, molecular context, antigen presentation time, etc.) are similar to those occurring during real infection process. In this regard, additional components used together with a vaccine antigen such as delivery systems (to ensure effective penetration through the mucosal barrier and increased concentration and stability of local antigen persistence) and adjuvants (their ability to trigger immune reactions artificially reconstructs events of pathogenic evasion) are much more important for mucosal delivery than for parenteral vaccines. Despite the unfading interest and decades of extensive research in this area only a few of mucosal vaccines are currently licensed [27]. Interestingly, while insufficient progress was explained generally by the lack of appropriate adjuvants (cholera toxin B subunit (CTB) being the only licensed component with adjuvant properties used in mucosal vaccines) it seems that the solution to developing an effective and safe mucosal vaccine has already been found and clinically proved [28].

Seven out of the all nine (78%) mucosal vaccines approved for use in humans (eight oral and one intranasal vaccine) are live-attenuated vaccines (LAV) exploiting the naturally acquired high immunogenic properties and mucosal tropism for effective antigen delivery. Looking precisely at intranasal vaccines against COVID-19, we could see that six out eight (75%) vaccines are based either on LAV or replicating (VVr) or non-replicating viral vectors (VVnr) compared to only 17 (12%) LAV- or viral vector-based COVID-19 vaccines among 142 for parenteral use. Notably, the two remaining intranasal subunit vaccines (Razi cov pars and CIGB-669) are used in three-dose regimen in order to improve immunogenicity, whereas among 46 parenteral subunit vaccines the vast majority (43 or 93%) have two- or even one-dose regimen. Such a drastic disproportion supports the idea that vaccines based on viral vectors or attenuated pathogens are the most suitable platforms for mucosal administration. The results of the present study aiming to evaluate the immunogenicity of Sputnik V vaccine based on two replication incompetent adenoviruses expressing S protein of SARS-CoV-2 (Ad6-S and Ad5-S) might be considered as an additional proof for the above statement.

In line with the common knowledge about mucosal vaccines, the intranasal Sputnik V administration induced both systemic and local immune response in mice and common marmosets, whereas intramuscular administration using the same vaccine and dose failed to form IgA antibodies in the respiratory tract in the studied animals [29]. Taking into account that IgA class switching occurs in nasal-associated lymphoid tissue (NALT) and there is a more than fourfold difference in body volume to nasal mucosa surface ratio between rodents and primates, the observed significant increase in antigen-specific respiratory IgA response both in mice and in common marmosets is of particular importance [18]. Contrary to intramuscular vaccines that were shown to be highly protective without inducing robust local IgA antibodies, this component of local immunity seems to be indispensable for intranasal vaccines [30]. Although the immune correlates of protection against COVID-19 might differ for intranasal and parenteral vaccines, the neutralizing activity of the sera appeared to be a reliable predictive marker of protectivity against symptomatic COVID-19 [9]. Having shown that intranasal vaccination with Sputnik V significantly elevated the neutralizing activity of the sera in both animal species, we surprisingly found no correlation between NtAb and anti-RBD IgG antibodies, which has been previously shown for intramuscular immunization and is widely used for diagnostic aims [31]. Interestingly, we detected a strong correlation between the anti-RBD IgG2a subtype and NtAb levels in mouse serum after intranasal vaccination, which was even stronger than after intramuscular vaccination. Therefore, we can speculate that the immunization route might influence the effector functions of IgG antibodies; however, this suggestion deserves to be thoroughly investigated. The observed correlation also has diagnostic value, with a perspective to develop ELISA tests that could evaluate a reliable immune correlate of protection upon intranasal vaccination.

One of the main drawbacks generally attributed to vector vaccines based on replication-incompetent adenoviruses is that their immunogenicity could be reduced by pre-existing anti-vector immunity acquired after natural infection or vaccination using the same vector. A number of effective strategies have been developed so far and shown to be effective to circumvent this phenomenon, such as utilizing different types of adenoviral vectors or different routes of vaccination, etc. [32]. Thus, in our previous studies, we used the heterologous prime-boost immunization scheme utilizing different types of adenoviral vectors and revealed no significant negative correlation between the pre-existing immune response to adenoviral vectors and neutralization titres in sera of volunteers vaccinated with Sputnik V [20]. The present study provides evidence that use of different vaccination routes might also be effective for Sputnik V. By analogy to local antigen-specific IgA antibodies, intramuscular vaccination with Sputnik V does not result in formation of anti-adenoviral IgA antibodies on the respiratory mucosa, thus indicating that intranasal vaccination with Sputnik V will not be compromised by previous intramuscular immunization (Supplementary Figure 2). Taking into account that more than half of Russian population have received a intramuscular injection of Sputnik V and that the revaccination is highly relevant today, the latter observation indicates that intranasal administration of Sputnik V might be a good choice for boosting the anti-SARS-CoV-2 immunity [33].

Overall, the present article demonstrates that intranasal prime-boost heterologous administration of Sputnik V is well-tolerated and effective for developing both local and systemic immune responses, as well as providing protection against lethal virus challenge, which is comparable to that of the initially developed intramuscular vaccine used here as a golden standard.

The results of this study have laid the basis for conducting a phase 1–2 clinical trial aiming at evaluating Safety, Tolerability and Immunogenicity of Gam-COVID-Vac Vaccine in a Nasal Spray (SPRAY) (NCT05248373). This trial, involving 400 participants either with or without pre-existing immunity to COVID-19 (formed after natural infection or vaccination) could also help to investigate whether intranasal vaccination with Sputnik V vaccine could be a good choice for revaccination providing robust mucosal and systemic immune response to SARS-CoV-2, including in persons having the pre-existing immunity to rAd5 and rAd26 vectors.

## Materials and methods

### Animal studies

C57BL/6 female SPF mice (4–5 weeks old) were procured from an onsite animal breeding facility. Transgenic hemizygous female and male K18-hACE2 mice (B6.Cg-Tg(K18-ACE2)2Prlmn/J; 4–5 weeks old) congenic on a C57BL/6 genetic background were initially purchased from the Jackson Laboratory (USA) and subsequently bred according to the official breeding considerations and following the General Terms and Conditions policy. Mice were housed in ventilated ISOCage P and N systems (Techniplast, Italy) for immunological and infection (BSL3 conforming) studies, respectively, with free access to autoclaved drinking water and standard chow diet. All of the experimental procedures were conducted in N.F.Gamaleya National Research Center for Epidemiology and Microbiology in compliance with the Guide for the Care and Use of Laboratory Animals (NIH Publication #85–23, revised 1996), and approved by the local animal ethics committee (protocol #9, 16 Apr 2021) and conducted.

The NHP studies were carried out at the Chumakov Federal Scientific Center for Research and Development of Immune and Biological Products, Russian Academy of Sciences, using healthy adult 2–6-year-old common marmosets (*Callithrix jacchus*) bred and raised in captivity under BSL-2 conditions at the onsite experimental clinic of *Callithricidae*. Primates were kept according to the EU Directive 2010/63/EU and Russian sanitary rules for experimental animal clinics (1045-73). The study protocol was approved by the Ethics Committee of Scientific Center (protocol #141021-2, 14 Oct 2021). Animals were identified by subcutaneous radiotags (Globalvet, Moscow). The Animal IDs in tables and graphs represent the last four digits of the tag ID.

Body weight of the primates was determined on days 0, 3, 7, 11, 15, 17, 24, 28, 31 and 35 after the first immunization using a Pioneer PA4102 electronic balance (Ohaus, USA). Body temperature of the animals was determined rectally (at a constant depth of 2 cm) the same days as body weight using an electronic thermometer DT-622 (A&D Company, Japan). General health condition parameters of the animals were assessed by a veterinarian daily starting from the day before the first immunization. Each abnormal observation was scored according to Supplementary Table S7 and the humane endpoint was set at 15 points. If the animal’s condition was to be scored at 15 points or above, humane euthanasia would be performed by anaesthesia overdose; death was confirmed by exsanguination, registration of the absence of breathing for 10 min and rigour mortis. No such cases occurred during the experiment.

### Immunization and further sampling procedures

The same formulation of prime-boost two-component Sputnik V vaccine (Gam-COVID-Vac) consisting of recombinant adenovirus type 26 (component 1 for prime vaccination) and recombinant adenovirus type 5 (component 2 for boosting) containing the SARS-CoV-2 S protein gene (rAd26-S and rAd5-S) in the amount of 10^11^ vp of each component was used for intramuscular (IM) and intranasal (IN) administration.

Mice were given two IM or IN injections of either 1/100 or 1/1000 of human dose of Sputnik V vaccine (10^9^vp or 10^8^vp) with a 3-week interval. The minimal volume of Sputnik V vaccine (5 µl) that was shown not to be able to reach the mouse lower respiratory tract upon intranasal delivery was used for immunizations [18,19].

The health status of common marmosets was examined by veterinarian and laboratory tests (normal parameters of CBC and biochemistry, absence of anti-adenovirus (Ad) 5/26 and anti-SARS-CoV-2 antibodies). A total of 12 primates (five males and seven females) with confirmed health status were randomized (four animals/group) and assigned for two vaccination with a 24-day interval. The first group was intramuscularly injected with 1/5 of the human dose (2 × 10^10^vp, 100µl) of Sputnik V vaccine into the femoral muscles. The second group was inoculated intranasally with 50 µL of the Sputnik V vaccine into each nostril an automatic pipette (a total of 2 × 10^10^vp). On the same days, placebo group animals were injected with the same volume of sterile isotonic saline preparation via both the IN and IM routes.

Mice were bled on day 14, 21 and 28 after first immunization to record kinetics of anti-RBD IgG and IgA antibodies in serum. On endpoint days mice were sacrificed using overdose of inhaled CO_2_. BAL specimens were obtained via trachea puncture using a 23-gauge needle catheter and infusing 0.5 mL of PBS into the lungs, followed by aspiration of this fluid into a syringe. Nasal lavage (NL) was performed using 0.5 mL of PBS, which was flushed through the trachea towards nares in a 1.7-mL microtubes. The tubes were placed in ice and sonicated for 20 min in ultrasonic water bath (Elma, Germany). Then, samples were centrifuged for 10 min at 1800 rpm at 4°C. The obtained BAL samples were immediately aliquoted and stored at −80°C until analysis.

Marmoset blood samples were collected from restrained conscious animals by femoral vein puncture into EDTA-containing tubes. Sample volume obtained from each animal did not exceed 4% of circulating blood volume per week. Nasal swabs were collected using type A human urinogenital probes (Polimernye Izdeliya Company, Russia) placed in 0.1 ml PBS containing proteinase inhibitor cocktail (Sigma-Aldrich) and 0.1% m/w sodium azide. Blood and nasal swab sampling was carried out at time points indicated in Figure 4(A) in a separate room with the exclusion of visual and auditory contact with other animals. The procedures did not cause any significant distress to the animals due to the preceding training.

### Biochemical and haematological measurements in marmoset blood

To evaluate the biochemical and haematological parameters blood samples (500 μL) were taken from each animal from each study group before the vaccine administration on days 0, 11, 24, and 31 after the first immunization. Biochemical analysis of serum was carried out using a Cobas c 111 automated biochemical analyzer (Roche, Switzerland) and the following liquid reagent kits: Alanine aminotransferase (ALTL), Aspartate aminotransferase (ASTL), Alkaline Phosphatase (ALP2S), Total Protein (TP2), Albumin (ALB2), Urea (UREAL), Triglycerides (TRIGL), Creatinine (CREJ2), General Bilirubin (BILT3), Bilirubin Direct (BILD2) as well as respective calibration and control kits according to the manufacturer’s instructions.

Quantitative and relative analysis of white blood cell populations (complete blood count, CBC) was performed manually in the Goryaev chamber.

### Cell lines and viruses

Vero E6 (ATCC CRL-1586) cells were maintained in Dulbecco’s modified Eagle’s medium (DMEM, HyClone, Cytiva, USA), supplemented with 10% or 2% of heat-inactivated fetal bovine serum (FBS, Capricorn Scientific, Germany), L-glutamine (4 mM) and penicillin/streptomycin solution (100 IU/mL; 100 μg/mL) (PanEco, Moscow, Russia).

Cells from mouse spleen and lungs as well as marmoset PBMCs were seeded in complete Roswell Park Memorial Institute (RPMI) medium supplemented with 10% heat-inactivated FBS (Gibco, USA).

The SARS-CoV-2 strain B.1.1.1 or PMVL-1 (GISAID EPI_ISL_421275) and B.1.617.2 (Delta) initially isolated from a nasopharyngeal swab was obtained from the State Collection of Viruses of the Gamaleya Center in Moscow and used in both challenge and titration of NtAbs studies. Isolation and further propagation were performed in Vero E6 cells in DMEM (HyClone Cytiva, Austria) with 2% heat-inactivated FBS (Capricorn Scientific GmbH, Germany): the cells were infected at multiplicity of infection (MOI) = 0.01 and incubated at 37°C in 5% CO_2_. The culture medium was collected at 72 h and clarified by centrifugation at 9000 g for 10 min at +4°C. The culture medium containing the virus was aliquoted, frozen and stored at −80°C.

### Evaluation of antibody titres in biological samples

Pre-coated 96-well plates from the ELISA kit developed at the Gamaleya National Research Center for Epidemiology and Microbiology and registered for clinical use in Russia (P3H 2020/10393 2020-05-18) were used for SARS-CoV-2 RBD-specific IgG analysis. For evaluation of anti-spike (S) antibodies SARS-CoV-2 S protein (S1 + S2 ectodomain) (Sino biological, YP_009724390.1) solution was used for coating 96-well plates (100 ng/well). Murine antigen-specific antibodies in the obtained samples were detected using anti-mouse total IgG, IgA and IgG1, IgG2a, IgG2b and IgG3 subtype-specific secondary HRP-conjugated antibodies (all of those were used in 1:5000 dilution) purchased from Abcam (Great Britain). Anti-monkey IgG (Sigma Aldrich, USA, 1:20,000 dilution) and anti-human IgA (was obtained as a gift from Dr. Marina Samoyovich, 1:40,000 dilution) secondary antibodies, both conjugated to HRP, were used to evaluate the humoral response of marmosets to vaccination. The ELISA protocol was used as published earlier [20]. A colorimetric signal was measured at 450 nm using a Multiscan FC spectrophotometric plate reader (Thermo Fisher Scientific, Waltham, MA, USA) 30 min after the addition of stop solution (4 M H_2_SO_4_) had been added.

### Neutralization assay with live SARS-CoV-2

Samples were inactivated by incubation at 56°C for 30 min prior to twofold serial dilution in complete DMEM supplemented with 2% heat-inactivated FBS. The samples were then mixed at a 1:1 ratio with 100TCID50 (50% tissue culture infectious dose) of SARS-CoV-2 in total volume of 100 μl and incubated at 37°C for 1 h. After that, antibody-virus complexes were added to Vero E6 cell monolayer and incubated for 96 h. The cytopathic effect (CPE) of the virus on the cell was assessed visually. Neutralization titre was defined as the highest serum dilution without any CPE in two of three replicable wells. Samples with no neutralization at starting dilution points were attributed to twofold lower values.

### Lymphocyte proliferation assay

Murine spleens were collected at the endpoint day and splenocytes were purified by density gradient centrifugation (400 g, 30 min) using Ficoll 1.09 g/mL (PanEco, Moscow, Russia). NHP PBMCs were isolated from whole blood by density gradient centrifugation (900 g, 30 min) using LSM (MP Biomedicals, USA). Splenocytes and PBMCs were washed in PBS, counted and stained using CellTrace™ carboxyfluorescein diacetate succinimidyl ester (CFSE) Cell Proliferation Kit (Invitrogen, USA) according to the previously described protocol [34]. Cells were seeded in duplicates in 96-well plates at 2 × 10^5^ cells per well and stimulated with 5 µg/ml SARS-CoV-2 S protein (S1 + S2 ectodomain) (Sino biological, YP_009724390.1). Cells and cell-free media samples were collected 96 hrs after treatment. Cells were stained with DAPI (1 µg/mL) to exclude dead cells and anti-CD3, anti-CD4, and anti-CD8 antibodies for 40 min at 4°C in Staining Buffer (BD Biosciences, USA). List of antibodies is provided in Supplementary Table S8. Proliferating CD4+ or CD8+ T-lymphocytes were expressed as a percentage of cells in the final culture that divided at least once (referring to “Fraction diluted” statistic) [35]. Cytokine analysis was performed in cell-free media samples using a mouse 23-plex bead-based Bio-Plex Pro Kit (Bio-Rad Laboratories, USA) and Non-Human Primate Th 14-Plex ProcartaPlex Panel kit (Thermo Scientific, USA) according to manufacturer's instructions.

### Cell-mediated immune response in the lungs

To discriminate between tissue-resident and blood-borne immune cells in the murine lungs three minutes before CO_2_ inhalation mice were injected intravenously with anti-CD45.2-FITC antibodies (10 mg/ml) diluted in sterile PBS as described previously [17].

The lungs were isolated, washed in PBS and transferred into gentle MACS C tubes containing buffer and enzymes from a Lung Dissociation Kit (Miltenyi Biotec GmbH, Germany). Lung tissue was digested according to the manufacturer’s instructions using the gentleMACS Octo dissociator (Miltenyi Biotec GmbH, Germany). Cells were seeded into 48-well plates at 2 × 10^6^ cells per well and stimulated with 5 µg/ml SARS-CoV-2 S protein (Sino biological, YP_009724390.1). Both antigen-stimulated and unstimulated cells were treated with 1 µg/ml anti-mouse CD28 and CD49d antibodies (both BD Biosciences, USA). Positive control cells were stimulated with 1 µg/ml ionomycin and 100 ng/ml phorbol 12-myristate 13-acetate (PMA, both Invivogen, USA). 1 µl of GolgiPlug^tm^ (BD Biosciences, USA) was added to each well after 1 h of incubation. 5 h later, the cells were placed at 4°C. After overnight storage the cells were washed in FACSbuffer and stained using Zombie Violet™ Fixable Viability Dye (FVD, Biolegend, USA), anti-CD45, anti-CD4, anti-CD8, anti-CD4 antibodies for 40 min at 4°C in Staining Buffer. The list of antibodies is provided in Supplementary Table S9. The cells were washed in Staining buffer and permeabilized using a Fixation/Permeabilization Solution Kit (both, BD Biosciences, USA) according to the manufacturer’s instructions. Anti-IL-17 and anti-IFNɣ antibodies were used for intracellular staining. Fluorescence Minus One (FMO) controls were used to set gates for positive and negative cytokine expression. Analysis was performed using a FACSAriaIII cytometer and FACSDiva and FlowJo Software (all from BD Biosciences, USA).

### Mouse challenge and tissue collection

Seven days after the second immunization K18-hACE2 mice (10mice/group) were stupefied by ether and challenged intranasally with 10^5^ TCID_50_ SARS-CoV-2 (B.1.1.1). Three animals from each group were euthanized by CO_2_ overdose at day 5 post infection for performing histological analysis of lung damages and for determining the viral load in the lungs (dissection of right interior lobe). All other animals in groups (7mice/group) were daily monitored for weight loss and survival. Euthanasia decision was made according to animal welfare scoring and humane endpoints.

### Lung viral load analysis, confirmation by PCR

Lung homogenates were prepared in DMEM (HyClone Cytiva, Austria) with 2% heat-inactivated FBS (Capricorn Scientific GmbH, Germany) using an MPbio FastPrep-24 homogenizer (MP Biomedicals LLC, USA). After homogenization, the suspension was centrifuged at 12,000 g at +4°C, the supernatant was used to determine the viral load. The infectious virus titre was determined on Vero E6 cells using a 50% tissue culture infectious dose (TCID_50_) assay. Serial tenfold dilutions of virus-containing samples were prepared in DMEM supplemented with 2% heat-inactivated FBS and added in 100 µl volume to Vero E6 cells in a 96-well plate in 4 replicates. The cells were incubated at 37°C in 5% CO_2_ for 96 hrs, and the cytopathic effect (CPE) was determined visually. The TCID50 titre was calculated according to the Reed–Muench method [36].

Extract RNA reagent (Evrogen, Russia) was used for total RNA isolation according to the manufacturer’s protocol. A POLYVIR SARS-CoV-2 real-time PCR kit (Lytech LLC, Russia) was used for PCR according to the manufacturer's protocol. A series of tenfold diluted virus RNA samples with a known concentration of GC/ml was used for quantitative genome copies (GC)/ml analysis. qPCR was performed on a CFX96 Thermocycler (Bio-Rad).

### Histopathology

Lungs from each mouse were fixed in 10% neutral buffered formalin (Soluform^tm^, JLS-Chemical, Russia) at +4°C, dehydrated in isoprepe (BioVitrum, Russia) using Microm STP 120 (Thermo Scientific, USA), and embedded in HISTOMIX (BioVitrum, Russia) using HistoStar workstation (Thermo Scientific, USA). 5-μm thick sections (10 per mouse) were cut using a Finesse ME+ microtome (Thermo Scientific, USA), stained with haematoxylin and eosin and mounted in Vitrogel (all BioVitrum, Russia). Pictures were obtained either using an Epson Perfection V600 scanner (9600 dpi) or using an Imager. Z1 microscope with an AxioCam MRc 5 camera (all ZEISS, Germany) at 20х and 63х magnification. Pathological changes were assessed using three parameters: (1) the acute lung injury (ALI) score, showing mostly the immunopathology in the lung parenchyma (from 0 to 1); (2) the peribronchiolar infiltration score, estimating the degree of inflammation of the tissue around the bronchioles (from 0 to 5); and (3) the perivascular infiltration score, estimating the degree of inflammation of the tissue around vessels (from 0 to 5). The exact parameters of tissue scoring systems are provided in the Supplementary Materials (p. 9).

### Statistical analysis

Statistical assessment of the differences in the studied parameters of general health, body temperature, body weight, biochemical analysis and complete blood count of the animals was performed using 2-way ANOVA with the Greenhouse-Gressier correction followed by Šidák multiple comparisons test in GraphPad Prism 9 software (v. 9.3.1, GraphPad, USA). The normality of data distribution was analyzed using the Shapiro-Wilks test. Depending on the normality, t-test or the Wilcoxon test was used for analysis of paired samples, t-test or the Mann–Whitney test – for unpaired samples, Pearson test or Spearman correlation test, for correlation analysis. Differences were considered significant at *p* <0.05.

## Supplementary Material

Supplemental Material
