# Supplementary material for: Immunogenicity and protectivity of intranasally delivered vector-based heterologous prime-boost COVID-19 vaccine Sputnik V in mice and non-human primates
Source: Emerg Microbes Infect. 2022 Sep 26;11(1):2229–47. doi: 10.1080/22221751.2022.2119169 (PMC9518644; doi:10.1080/22221751.2022.2119169)
Supplement: Supplemental Material [file TEMI_A_2119169_SM1307.docx]

**Supplementary information**

**Immunogenicity and protection of intranasally delivered vector-based heterologous prime-boost COVID-19 vaccine Sputnik V in mice and nonhuman primates**

**Table S1 –** **Spearman test for determination of correlation between RBD-specific antibodies in serum as well as in BAL samples and NtAb in serum samples 21 days after intramuscular (IM) or intranasal (IN) vaccination.** For each pair correlation coefficient (r) and the p-value (p) are presented. Pairs with strong (r>0.8) correlation indicated in bold.

|  | Group | r | p |
| --- | --- | --- | --- |
| IM (n=7) | Anti-RBD IgG in serum vs NtAb | **0.9873** | **<0.0001** |
|  | Anti-RBD IgA in serum vs NtAb | -0.1164 | 0.8037 |
|  | Anti-RBD IgG in BAL vs NtAb | 0.5256 | 0.2257 |
|  | Anti-RBD IgA in BAL vs NtAb | No IgA in BAL | No IgA in BAL |
| IN (n=7) | Anti-RBD IgG in serum vs NtAb | -0.2188 | 0.6374 |
|  | Anti-RBD IgA in serum vs NtAb | -0.06644 | 0.8875 |
|  | Anti-RBD IgG in BAL vs NtAb | -0.3003 | 0.5129 |
|  | Anti-RBD IgA in BAL vs NtAb | -0.3179 | 0.4872 |

**Table S2 – Spearman test for determination of correlation between RBD-specific IgG subclasses in serum and NtAb in serum samples 21 days after IM or IN vaccination.** For each pair correlation coefficient (r) and the p-value (p) are presented. Pairs with strong (r>0.8) correlation indicated in bold.

|  | Group | r | p |
| --- | --- | --- | --- |
| IM (n=7) | Anti-RBD IgG1 in serum vs VNA | **0.8436** | **0.0170** |
|  | Anti-RBD IgG2a in serum vs VNA | **0.8060** | **0.0286** |
|  | Anti-RBD IgG2b in serum vs VNA | 0.4092 | 0.3620 |
|  | Anti-RBD IgG3 in serum vs VNA | **0.8933** | **0.0067** |
| IN (n=7) | Anti-RBD IgG1 in serum vs VNA | -0.3560 | 0.4331 |
|  | Anti-RBD IgG2a in serum vs VNA | **0.9083** | **0.0047** |
|  | Anti-RBD IgG2b in serum vs VNA | 0.04551 | 0.9228 |
|  | Anti-RBD IgG3 in serum vs VNA | -0.2360 | 0.6103 |

**Table S3 – General welfare score of common marmosets (Callithrix jacchus) in groups receiving adenovirus SARS-CoV-2 vaccine via IN and IM route and in control group**

| **Days** | **General condition, points** | | | | | | | | | | | | | |
| --- | --- | --- | --- | --- | --- | --- | --- | --- | --- | --- | --- | --- | --- | --- |
|  | **Animal ID, sex** | | | | | | | | | | | | | |
|  | **Placebo group** | | | | **Intramuscular injection** | | | | | **Intranasal administration** | | | | |
|  | 0728 M | 2585 F | 0727 F | 0708 M | 0722 M | 0729 F | 0701 F | 0703 F | **P*** | 3018 M | 0704 F | 0724 M | 2584 F | **P*** |
| **-1** | 0 | 0 | 0 | 0 | 1 | 0 | 0 | 0 | ns | 0 | 1 | 0 | 0 | ns |
| **0** | 1 | 0 | 1 | 1 | 1 | 1 | 0 | 1 | ns | 0 | 1 | 0 | 0 | ns |
| **1** | 1 | 0 | 0 | 0 | 1 | 1 | 0 | 1 | ns | 0 | 1 | 0 | 0 | ns |
| **2** | 1 | 1 | 0 | 0 | 1 | 0 | 0 | 1 | ns | 0 | 1 | 1 | 1 | ns |
| **3** | 0 | 0 | 0 | 0 | 0 | 0 | 1 | 0 | ns | 0 | 0 | 0 | 0 | n/a |
| **4** | 0 | 0 | 0 | 0 | 0 | 0 | 1 | 0 | ns | 0 | 0 | 0 | 0 | n/a |
| **5** | 1 | 0 | 0 | 0 | 1 | 0 | 1 | 1 | ns | 0 | 1 | 0 | 0 | ns |
| **6** | 1 | 0 | 0 | 1 | 1 | 0 | 0 | 1 | ns | 0 | 1 | 0 | 0 | ns |
| **7** | 0 | 0 | 0 | 0 | 0 | 1 | 0 | 0 | ns | 0 | 0 | 0 | 0 | n/a |
| **8** | 1 | 0 | 0 | 0 | 1 | 0 | 0 | 1 | ns | 0 | 1 | 0 | 0 | ns |
| **9** | 0 | 0 | 0 | 0 | 0 | 1 | 1 | 0 | ns | 0 | 0 | 0 | 0 | n/a |
| **10** | 1 | 0 | 0 | 0 | 0 | 0 | 0 | 1 | ns | 0 | 0 | 0 | 0 | ns |
| **11** | 1 | 0 | 0 | 1 | 1 | 1 | 1 | 1 | ns | 0 | 1 | 0 | 1 | ns |
| **12** | 0 | 0 | 0 | 0 | 1 | 0 | 0 | 0 | ns | 0 | 1 | 0 | 0 | ns |
| **13** | 0 | 0 | 0 | 0 | 1 | 0 | 0 | 0 | ns | 0 | 1 | 0 | 0 | ns |
| **14** | 0 | 0 | 0 | 0 | 0 | 0 | 1 | 0 | ns | 0 | 0 | 0 | 0 | ns |
| **15** | 1 | 0 | 0 | 0 | 0 | 0 | 1 | 1 | ns | 0 | 0 | 0 | 0 | ns |
| **16** | 1 | 0 | 0 | 0 | 0 | 0 | 0 | 1 | ns | 0 | 0 | 0 | 0 | ns |
| **17** | 0 | 0 | 0 | 0 | 0 | 1 | 0 | 0 | ns | 0 | 0 | 0 | 0 | n/a |
| **18** | 0 | 0 | 0 | 0 | 0 | 0 | 1 | 0 | ns | 0 | 0 | 0 | 0 | n/a |
| **19** | 1 | 0 | 0 | 0 | 1 | 0 | 0 | 1 | ns | 0 | 1 | 0 | 0 | ns |
| **20** | 1 | 0 | 0 | 1 | 1 | 0 | 0 | 1 | ns | 0 | 1 | 0 | 0 | ns |
| **21** | 1 | 0 | 0 | 0 | 0 | 0 | 1 | 1 | ns | 0 | 0 | 0 | 0 | ns |
| **22** | 0 | 0 | 0 | 0 | 0 | 0 | 1 | 0 | ns | 0 | 0 | 0 | 0 | n/a |
| **23** | 1 | 0 | 0 | 0 | 0 | 0 | 0 | 1 | ns | 0 | 0 | 0 | 0 | ns |
| **24** | 0 | 0 | 0 | 0 | 1 | 1 | 1 | 0 | ns | 0 | 1 | 0 | 0 | ns |
| **25** | 0 | 1 | 0 | 0 | 1 | 0 | 1 | 0 | ns | 0 | 1 | 1 | 1 | ns |
| **26** | 0 | 0 | 0 | 0 | 0 | 1 | 1 | 0 | ns | 0 | 0 | 0 | 0 | n/a |
| **27** | 2 | 0 | 0 | 0 | 1 | 0 | 1 | 2 | ns | 0 | 1 | 0 | 0 | ns |
| **28** | 1 | 0 | 0 | 0 | 0 | 0 | 1 | 1 | ns | 0 | 0 | 0 | 0 | ns |
| **29** | 0 | 0 | 0 | 0 | 0 | 1 | 1 | 0 | ns | 0 | 0 | 0 | 0 | n/a |
| **30** | 0 | 0 | 0 | 0 | 0 | 1 | 1 | 0 | ns | 0 | 0 | 0 | 0 | n/a |
| **31** | 1 | 0 | 1 | 0 | 1 | 2 | 1 | 0 | ns | 0 | 2 | 0 | 0 | ns |
| **32** | 1 | 0 | 0 | 0 | 1 | 0 | 1 | 1 | ns | 0 | 1 | 0 | 0 | ns |
| **33** | 1 | 1 | 1 | 0 | 1 | 0 | 1 | 1 | ns | 0 | 1 | 1 | 1 | ns |
| **34** | 0 | 1 | 0 | 0 | 1 | 1 | 1 | 0 | ns | 1 | 1 | 1 | 1 | ns |
| **35** | 0 | 1 | 0 | 1 | 1 | 1 | 1 | 0 | ns | 1 | 1 | 1 | 1 | ns |

* — pairwise comparison vs control group in RM two-way ANOVA with Geisser-Greenhouse correction and Šidák multiple comparisons test; ns — no statistically significant difference (P>0.05); n/a — statistical analysis not applicable due to null values.

**Table S4 – Body weight of common marmosets (Callithrix jacchus) in groups receiving adenovirus SARS-CoV-2 vaccine via IN and IM route and in control group**

| **Days** | **Body weight, grams** | | | | | | | | | | | | | |
| --- | --- | --- | --- | --- | --- | --- | --- | --- | --- | --- | --- | --- | --- | --- |
|  | **Animal ID, sex** | | | | | | | | | | | | | |
|  | **Placebo group** | | | | **Intramuscular injection** | | | | | **Intranasal administration** | | | | |
|  | 0728 M | 2585 F | 0727 F | 0708 M | 0722 M | 0729 F | 0701 F | 0703 F | **P*** | 3018 M | 0704 F | 0724 M | 2584 F | **P*** |
| **0** | 420 | 400 | 400 | 490 | 390 | 370 | 380 | 480 | ns | 400 | 490 | 350 | 420 | ns |
| **3** | 420 | 380 | 370 | 460 | 380 | 380 | 380 | 470 | ns | 390 | 480 | 370 | 405 | ns |
| **7** | 420 | 390 | 360 | 450 | 390 | 370 | 370 | 470 | ns | 390 | 470 | 370 | 400 | ns |
| **11** | 420 | 400 | 380 | 460 | 400 | 380 | 390 | 500 | ns | 410 | 480 | 380 | 420 | ns |
| **15** | 420 | 380 | 370 | 450 | 395 | 380 | 370 | 480 | ns | 410 | 490 | 370 | 400 | ns |
| **17** | 420 | 380 | 360 | 440 | 380 | 380 | 385 | 470 | ns | 405 | 495 | 380 | 405 | ns |
| **24** | 400 | 360 | 370 | 460 | 380 | 380 | 370 | 490 | ns | 420 | 500 | 380 | 400 | ns |
| **28** | 420 | 365 | 370 | 450 | 360 | 370 | 365 | 490 | ns | 415 | 480 | 370 | 400 | ns |
| **31** | 420 | 395 | 400 | 470 | 380 | 380 | 380 | 500 | ns | 430 | 490 | 370 | 420 | ns |
| **35** | 410 | 380 | 380 | 470 | 370 | 385 | 370 | 500 | ns | 410 | 500 | 370 | 400 | ns |

* — pairwise comparison vs control group in RM two-way ANOVA with Geisser-Greenhouse correction and Šidák multiple comparisons test; ns — no statistically significant difference (P>0.05).

**Table S5 – Body temperature of common marmosets (Callithrix jacchus) in groups receiving adenovirus SARS-CoV-2 vaccine via IN and IM route and in control group**

| **Days** | **Body temperature, ˚C** | | | | | | | | | | | | | |
| --- | --- | --- | --- | --- | --- | --- | --- | --- | --- | --- | --- | --- | --- | --- |
|  | **Animal ID, sex** | | | | | | | | | | | | | |
|  | **Placebo group** | | | | **Intramuscular injection** | | | | | **Intranasal administration** | | | | |
|  | 0728 M | 2585 F | 0727 F | 0708 M | 0722 M | 0729 F | 0701 F | 0703 F | **P*** | 3018 M | 0704 F | 0724 M | 2584 F | **P*** |
| **0** | 40.8 | 40.6 | 40.7 | 39.7 | 40.4 | 40.4 | 39.8 | 40.8 | ns | 39.4 | 39.9 | 40.4 | 40.6 | ns |
| **3** | 39.9 | 40.8 | 40 | 39.8 | 39.8 | 39.8 | 39.8 | 40.5 | ns | 39.8 | 40.3 | 40.3 | 40.6 | ns |
| **7** | 40.2 | 40.8 | 40.5 | 40 | 40.6 | 40.2 | 40.1 | 40.4 | ns | 39.8 | 40.8 | 41 | 40.5 | ns |
| **11** | 40.5 | 40.3 | 40.5 | 39.5 | 40.3 | 40.2 | 40.1 | 40.6 | ns | 39.6 | 40.6 | 40.5 | 40.5 | ns |
| **15** | 39.8 | 40.8 | 40.5 | 40 | 40.4 | 40 | 39.7 | 40.3 | ns | 40 | 40.5 | 40.5 | 41 | ns |
| **17** | 40.1 | 40.6 | 40.3 | 39.6 | 40.2 | 39.6 | 39.9 | 39.8 | ns | 39.5 | 40.2 | 41 | 40.5 | ns |
| **24** | 40.5 | 40.5 | 40.7 | 40 | 39.9 | 40 | 39.8 | 40.4 | ns | 39.9 | 39.5 | 40.4 | 40.7 | ns |
| **28** | 40.5 | 40.8 | 40.3 | 40.2 | 39.6 | 39.8 | 39.8 | 39.6 | **0.013** | 39.2 | 40.3 | 40 | 41 | ns |
| **31** | 40.7 | 39.9 | 40.3 | 39.5 | 40.1 | 40.2 | 40.1 | 40.4 | ns | 39.4 | 40.3 | 40.8 | 40.3 | ns |
| **35** | 40.3 | 40.8 | 40 | 40 | 40.1 | 39.5 | 40.1 | 39.7 | ns | 39.6 | 40 | 40.8 | 41 | ns |

* — pairwise comparison vs control group in RM two-way ANOVA with Geisser-Greenhouse correction and Šidák multiple comparisons test; ns — no statistically significant difference (P>0.05); statistically significant differences are outlined in bold.

**Table S6 – Biochemical and hematological blood parameters of common marmosets (Callithrix jacchus) in groups receiving adenovirus SARS-CoV-2 vaccine via IN and IM route and in control group. Reference parameters were applied as previously published.**

| **Days** | **Parameters** | | | | | | | | | | | | | | |
| --- | --- | --- | --- | --- | --- | --- | --- | --- | --- | --- | --- | --- | --- | --- | --- |
|  | **Animal ID, sex** | | | | | | | | | | | | | | |
|  | **Placebo group** | | | | **Intramuscular injection** | | | | | **Intranasal administration** | | | | | |
|  | 0728 M | 2585 F | 0727 F | 0708 M | 0722 M | 0729 F | 0701 F | 0703 F | **P*** | 3018 M | 0704 F | 0724 M | 2584 F | **P*** |  |
| **Alanine aminotransferase, U/L [REF. 0.79–45.33] [1]** | | | | | | | | | | | | | | | |
| **0** | 14.1 | 4.3 | 6.3 | 4.8 | 3.8 | 7.2 | 4.6 | 6.2 | ns | 8.7 | 6.7 | 6.7 | 8 | ns |  |
| **11** | 6.6 | 12.3 | 11.6 | 4.2 | 4.6 | 7.3 | 27 | 9.3 | ns | 4.5 | 9.5 | 56.6 | 7.8 | ns |  |
| **24** | 6.8 | 3.6 | 23.6 | 6.4 | 3.3 | 4.4 | 3.4 | 3.7 | ns | 3.5 | 4.6 | 5.6 | 7 | ns |  |
| **31** | 5.5 | 5.7 | 6.7 | 2.7 | 3.2 | 3.2 | 3.3 | 3 | ns | 2.3 | 6.1 | 5.1 | 12.8 | ns |  |
| **Aspartate aminotransferase, U/L [REF. 51.24–316.12] [1]** | | | | | | | | | | | | | | | |
| **0** | 198.3 | 114.6 | 172.9 | 140.6 | 107.3 | 143.6 | 109.2 | 135.8 | ns | 184.2 | 147.3 | 152.8 | 141.5 | ns |  |
| **11** | 131.9 | 141.7 | 140 | 102.9 | 100.9 | 133 | 233.1 | 127.7 | ns | 101 | 131.6 | 260.2 | 146.7 | ns |  |
| **24** | 133.3 | 115 | 141.8 | 147.6 | 128.9 | 127.9 | 107.9 | 127.2 | ns | 104.1 | 119.6 | 127.6 | 129.9 | ns |  |
| **31** | 139.5 | 130.8 | 132.4 | 108.3 | 114.6 | 89.8 | 107.8 | 97.4 | ns | 93.5 | 103.9 | 109.2 | 126.2 | ns |  |
| **Alkaline phosphatase , U/L [REF. 44.00–426.25] [1]** | | | | | | | | | | | | | | | |
| **0** | 158.3 | 159.4 | 154.2 | 89 | 112.1 | 98.3 | 121.6 | 129.7 | ns | 88.9 | 117 | 199 | 191.3 | ns |  |
| **11** | 120 | 115 | 121.7 | 85.7 | 107 | 90.2 | 125 | 127.4 | ns | 115 | 111.4 | 161.7 | 159.3 | ns |  |
| **24** | 125.4 | 140.2 | 113.6 | 108.1 | 134.4 | 105.6 | 122.8 | 131.5 | ns | 153.6 | 117.1 | 190.5 | 160.8 | ns |  |
| **31** | 127.8 | 137.8 | 137.7 | 96.5 | 131.3 | 97.2 | 104.8 | 126.6 | ns | 155.7 | 106.6 | 173.7 | 163.9 | ns |  |
| **Total protein, g/L [REF. 41.1–74.8] [1]** | | | | | | | | | | | | | | | |
| **0** | 70.4 | 69.7 | 76.4 | 73 | 70.9 | 70 | 68.3 | 72.5 | ns | 66.7 | 77.2 | 74.3 | 74.3 | ns |  |
| **11** | 72.1 | 70.5 | 71.8 | 73.6 | 70.9 | 68.7 | 76.6 | 73.4 | ns | 69.6 | 71 | 71.3 | 70.3 | ns |  |
| **24** | 74.5 | 70.2 | 72.9 | 76.3 | 73.9 | 75.3 | 73.6 | 73.2 | ns | 74 | 75.2 | 78.6 | 74.6 | ns |  |
| **31** | 72.2 | 69.2 | 77.5 | 78.3 | 72.9 | 66.9 | 68.9 | 75.2 | ns | 77.3 | 71 | 71.8 | 74.4 | ns |  |
| **Albumin, g/L [REF. 10.77–57.88] [1]** | | | | | | | | | | | | | | | |
| **0** | 50.8 | 50.62 | 51.46 | 45.81 | 48.48 | 47.06 | 43 | 49.12 | ns | 41.68 | 53.58 | 52.02 | 52.56 | ns |  |
| **11** | 48.68 | 45.51 | 47.02 | 44.58 | 45.75 | 42.29 | 45.03 | 47.82 | ns | 40.42 | 46.47 | 46.28 | 46.86 | ns |  |
| **24** | 51.75 | 49.26 | 49.59 | 46.96 | 49.23 | 49.87 | 45.65 | 49.12 | ns | 44.95 | 50.15 | 51.52 | 49.9 | ns |  |
| **31** | 49.75 | 47.4 | 50.86 | 46.57 | 45.8 | 43.76 | 42 | 49.9 | ns | 48.05 | 48.78 | 48.45 | 50.91 | ns |  |
| **Urea, mmol/L [REF. 3.37–18.67] [1]** | | | | | | | | | | | | | | | |
| **0** | 4.04 | 4.76 | 2.74 | 4.09 | 3.06 | 5.79 | 5.61 | 4.97 | ns | 5.22 | 7.79 | 3.36 | 5.56 | ns |  |
| **11** | 3.17 | 5.93 | 4.83 | 6.46 | 3.62 | 4.28 | 7.59 | 5.31 | ns | 3.98 | 5.45 | 3.93 | 7.14 | ns |  |
| **24** | 3.02 | 4.36 | 2.67 | 4.45 | 3.27 | 4.38 | 5.09 | 4.24 | ns | 2.21 | 6.23 | 3.35 | 6.56 | ns |  |
| **31** | 3.9 | 6.7 | 2.88 | 4.89 | 3.95 | 3.8 | 3.67 | 2.47 | ns | 2.96 | 4.54 | 1.98 | 4.42 | ns |  |
| **Triglycerides, mmol/L [REF. 0.56–2.63] [1]** | | | | | | | | | | | | | | | |
| **0** | 1.27 | 0.79 | 0.93 | 2.09 | 0.69 | 1.14 | 0.48 | 1.18 | ns | 2.17 | 1.39 | 1.48 | 1.08 | ns |  |
| **11** | 1.4 | 1.14 | 1.02 | 2.61 | 0.72 | 1.22 | 0.98 | 2.12 | ns | 2.98 | 2.25 | 1.85 | 1.54 | ns |  |
| **24** | 1.25 | 0.76 | 1.52 | 2.99 | 0.85 | 1.29 | 0.72 | 2.13 | ns | 1.36 | 3.89 | 1.47 | 3.18 | ns |  |
| **31** | 1.59 | 0.74 | 1.24 | 2.59 | 1.43 | 0.57 | 0.64 | 1.47 | ns | 1.08 | 2.26 | 1.16 | 0.8 | ns |  |
| **Creatinine, mmol/L [REF. 38.94–62.38] [2]** | | | | | | | | | | | | | | | |
| **0** | 57.8 | 49.7 | 49.3 | 41.9 | 50.9 | 52.8 | 39 | 44.7 | ns | 53.4 | 49.3 | 59.6 | 54.6 | ns |  |
| **11** | 46.3 | 51.8 | 56.5 | 38.6 | 51.1 | 49.9 | 45.2 | 48 | ns | 51 | 45.8 | 66.6 | 49.2 | ns |  |
| **24** | 45.8 | 57.3 | 48 | 45.2 | 54.4 | 49 | 44.6 | 39.2 | ns | 39.5 | 40.2 | 55.1 | 55.5 | ns |  |
| **31** | 53.3 | 49.9 | 49 | 37.6 | 54.1 | 47.3 | 39.1 | 45.2 | ns | 36.8 | 41.8 | 57.4 | 56.9 | ns |  |
| **Total bilirubin, umol/L [REF. 0.03–4.24] [1]** | | | | | | | | | | | | | | | |
| **0** | 0.2 | 0.9 | 0 | 0 | 0.6 | 0 | 0 | 0 | ns | 1.3 | 0 | 0.6 | 0.2 | ns |  |
| **11** | 0 | 0 | 0 | 0 | 1.3 | 0 | 0 | 0 | ns | 0 | 0 | 0 | 0 | ns |  |
| **24** | 0.2 | 0.2 | 0.2 | 0 | 0 | 0 | 0.6 | 0 | ns | 0 | 0 | 0.2 | 0.2 | ns |  |
| **31** | 0 | 0 | 0 | 0 | 0 | 0.2 | 0 | 0 | n/a | 0.2 | 0.2 | 0 | 0 | ns |  |
| **Direct bilirubin, umol/L [REF. 0.0–1.3] [2]** | | | | | | | | | | | | | | | |
| **0** | 0.5 | 0.6 | 0 | 0 | 0.2 | 0 | 0 | 0.1 | ns | 1 | 0.5 | 0.3 | 0.5 | ns |  |
| **11** | 0.1 | 0 | 0.1 | 0.1 | 0.4 | 0 | 0.1 | 0.1 | ns | 0.5 | 0.2 | 0.4 | 0.1 | ns |  |
| **24** | 0.1 | 0 | 0.1 | 0.5 | 0.5 | 0 | 0 | 0.3 | ns | 0 | 0 | 0 | 0.1 | ns |  |
| **31** | 0 | 0 | 0 | 0.4 | 0.4 | 0 | 0 | 0 | ns | 0.4 | 0.7 | 0 | 0.1 | ns |  |
| **Red blood cells, cells*10^12/L [REF. 3.47–7.88] [1]** | | | | | | | | | | | | | | | |
| **0** | 7.67 | 6.83 | 7.25 | 7.82 | 9.19 | 5.25 | 5.67 | 8.24 | ns | 5.83 | 9.14 | 8.66 | 7.61 | ns |  |
| **11** | 7.67 | 5.72 | 5.62 | 6.14 | 5.04 | 5.09 | 3.68 | 7.88 | ns | 7.35 | 6.83 | 7.77 | 5.51 | ns |  |
| **24** | 7.77 | 6.72 | 7 | 7.07 | 9.94 | 8.33 | 5.39 | 8.68 | ns | 6.16 | 9.31 | 7.14 | 7.77 | ns |  |
| **31** | 9.03 | 7.56 | 7.84 | 9.17 | 8.12 | 7.07 | 4.34 | 8.19 | ns | 7 | 10.78 | 7.28 | 6.3 | ns |  |
| **White blood cells, cells*10^9/L [REF. 2.72–10.85] [1]** | | | | | | | | | | | | | | | |
| **0** | 8.66 | 13.52 | 9.98 | 12.99 | 12.34 | 10.5 | 9.71 | 10.24 | ns | 11.16 | 5.78 | 8.27 | 14.18 | ns |  |
| **11** | 6.69 | 6.83 | 8.53 | 8.01 | 10.89 | 9.71 | 6.56 | 7.35 | ns | 13.91 | 9.32 | 4.99 | 6.96 | ns |  |
| **24** | 8.79 | 6.43 | 6.56 | 8.53 | 8.4 | 13.39 | 5.78 | 6.56 | ns | 11.29 | 4.99 | 8.01 | 13.26 | ns |  |
| **31** | 7.35 | 8.14 | 5.12 | 8.93 | 8.79 | 9.06 | 6.96 | 9.19 | ns | 9.71 | 5.91 | 6.43 | 7.74 | ns |  |
| **Band neutrophils, % [REF. 0.0–4.0] [1]** | | | | | | | | | | | | | | | |
| **0** | 0 | 0 | 0 | 2 | 1 | 3 | 2 | 1 | ns | 3 | 1 | 0 | 1 | ns |  |
| **11** | 2 | 0 | 1 | 2 | 2 | 1 | 0 | 1 | ns | 2 | 1 | 0 | 0 | ns |  |
| **24** | 0 | 0 | 0 | 5 | 2 | 1 | 0 | 0 | ns | 2 | 1 | 0 | 0 | ns |  |
| **31** | 3 | 0 | 1 | 1 | 1 | 0 | 2 | 1 | ns | 1 | 1 | 1 | 0 | ns |  |
| **Segmented neutrophils, % [REF. 28.25–78.05] [1]** | | | | | | | | | | | | | | | |
| **0** | 47 | 55 | 37 | 48 | 55 | 40 | 47 | 45 | ns | 67 | 48 | 67 | 55 | ns |  |
| **11** | 59 | 38 | 44 | 66 | 65 | 52 | 56 | 33 | ns | 46 | 44 | 71 | 82 | ns |  |
| **24** | 64 | 59 | 35 | 59 | 73 | 66 | 54 | 25 | ns | 44 | 50 | 71 | 75 | ns |  |
| **31** | 60 | 60 | 47 | 64 | 39 | 43 | 43 | 34 | **0.018** | 37 | 38 | 64 | 66 | ns |  |
| **Basophils, % [REF. 0.0–5.0] [3]** | | | | | | | | | | | | | | | |
| **0** | 1 | 1 | 0 | 0 | 1 | 0 | 1 | 0 | ns | 0 | 1 | 1 | 0 | ns |  |
| **11** | 0 | 0 | 0 | 0 | 0 | 1 | 0 | 0 | ns | 0 | 1 | 0 | 0 | ns |  |
| **24** | 1 | 1 | 0 | 0 | 3 | 1 | 1 | 0 | ns | 3 | 0 | 1 | 1 | ns |  |
| **31** | 0 | 0 | 0 | 1 | 2 | 7 | 0 | 1 | ns | 3 | 0 | 0 | 0 | ns |  |
| **Eosinophils, % [REF. 0.0–13.75] [1]** | | | | | | | | | | | | | | | |
| **0** | 0 | 0 | 0 | 0 | 0 | 1 | 1 | 0 | ns | 0 | 0 | 0 | 0 | n/a |  |
| **11** | 0 | 0 | 0 | 0 | 0 | 0 | 0 | 0 | n/a | 0 | 0 | 0 | 0 | n/a |  |
| **24** | 0 | 0 | 0 | 0 | 0 | 0 | 0 | 0 | n/a | 0 | 0 | 0 | 0 | n/a |  |
| **31** | 0 | 0 | 0 | 0 | 1 | 0 | 0 | 0 | ns | 0 | 0 | 0 | 0 | n/a |  |
| **Monocytes, % [REF. 2.0–17.05] [1]** | | | | | | | | | | | | | | | |
| **0** | 3 | 4 | 3 | 7 | 6 | 8 | 3 | 4 | ns | 4 | 3 | 3 | 6 | ns |  |
| **11** | 4 | 3 | 2 | 5 | 1 | 3 | 2 | 3 | ns | 3 | 5 | 2 | 3 | ns |  |
| **24** | 2 | 2 | 2 | 2 | 1 | 2 | 3 | 4 | ns | 5 | 2 | 3 | 2 | ns |  |
| **31** | 3 | 3 | 5 | 4 | 4 | 4 | 2 | 4 | ns | 3 | 4 | 2 | 2 | ns |  |
| **Lymphocytes, % [REF. 14.0–62.70] [1]** | | | | | | | | | | | | | | | |
| **0** | 49 | 40 | 60 | 43 | 37 | 48 | 46 | 50 | ns | 26 | 47 | 29 | 38 | ns |  |
| **11** | 35 | 59 | 53 | 27 | 32 | 43 | 42 | 63 | ns | 49 | 49 | 27 | 15 | ns |  |
| **24** | 33 | 38 | 63 | 34 | 21 | 30 | 42 | 71 | ns | 46 | 47 | 25 | 22 | ns |  |
| **31** | 34 | 37 | 47 | 30 | 53 | 46 | 53 | 60 | **0.029** | 56 | 57 | 33 | 32 | ns |  |

* — pairwise comparison vs control group in RM two-way ANOVA with Geisser-Greenhouse correction and Šidák multiple comparisons test; ns — no statistically significant difference (P>0.05); statistically significant differences are outlined in bold.

References

1. Kuehnel, F., Grohmann, J., Buchwald, U., Koeller, G., Teupser, D., & Einspanier, A. (2012). Parameters of haematology, clinical chemistry and lipid metabolism in the common marmoset and alterations under stress conditions. Journal of medical primatology, 41(4), 241-250.

2. Marini, R. P., Wachtman, L. M., Tardif, S. D., Mansfield, K., & Fox, J. G. (Eds.). (2018). The common marmoset in captivity and biomedical research. Academic Press.

3. Yarbrough, L. W., Tollett, J. L., Montrey, R. D., & Beattie, R. J. (1984). Serum biochemical, hematological and body measurement data for common marmosets (Callithrix jacchus jacchus). Laboratory animal science, 34(3), 276-280.

**Supplementary Figure 1 – Unsignificant cytokine production in marmoset PBMCs received from intramuscularly (IM) or intranasally (IN) vaccinated with Sputnik V vaccine over unvaccinated animals.** Cytokine data are presented as the difference (delta) in cytokine concentrations between samples with and without protein stimulation. Dots show individual data points. Each bar represents the mean value per group ± SD (error bars). Lower limit of quantification (LLOQ) is indicated by a gray line.


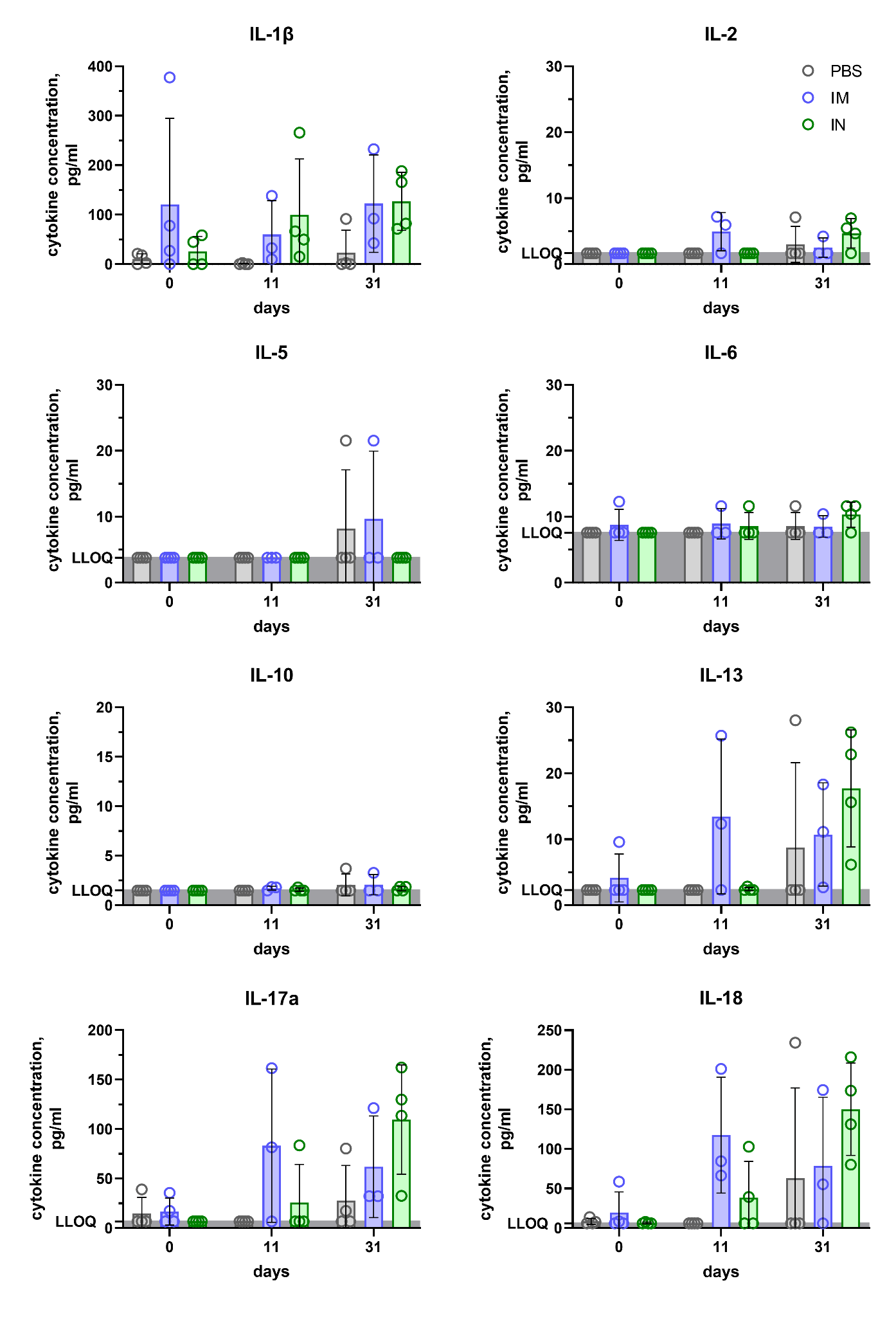


**Supplementary Figure 2 – Humoral immune response against adenoviral vectors (Ad26, Ad5) of common marmosets and mice received intramuscular (IM) or intranasal (IN) Sputnik V vaccine.**

Anti-Ad26 and anti-Ad5 IgA in BAL (A) in serum (B) as well as Anti-Ad26 and anti-Ad5 IgG in serum (C) of mice 42 days after receiving IM or IN Sputnik V vaccine. Anti-Ad26 and anti-Ad5 IgA in nasal swabs (D) in serum (E) as well as Anti-Ad26 and anti-Ad5 IgG in serum (F) of common marmosets 116 days after receiving IM or IN Sputnik V vaccine. Bars represent geometric mean for each group with 95%CI. Dots show individual data points. Significant differences between vaccinated and unvaccinated animals were calculated using two-way Mann-Whitney test (*p < 0.05). Significant differences between IN and IM vaccinated animals were calculated using Mann-Whitney test and indicated by hashes (### p < 0.005).


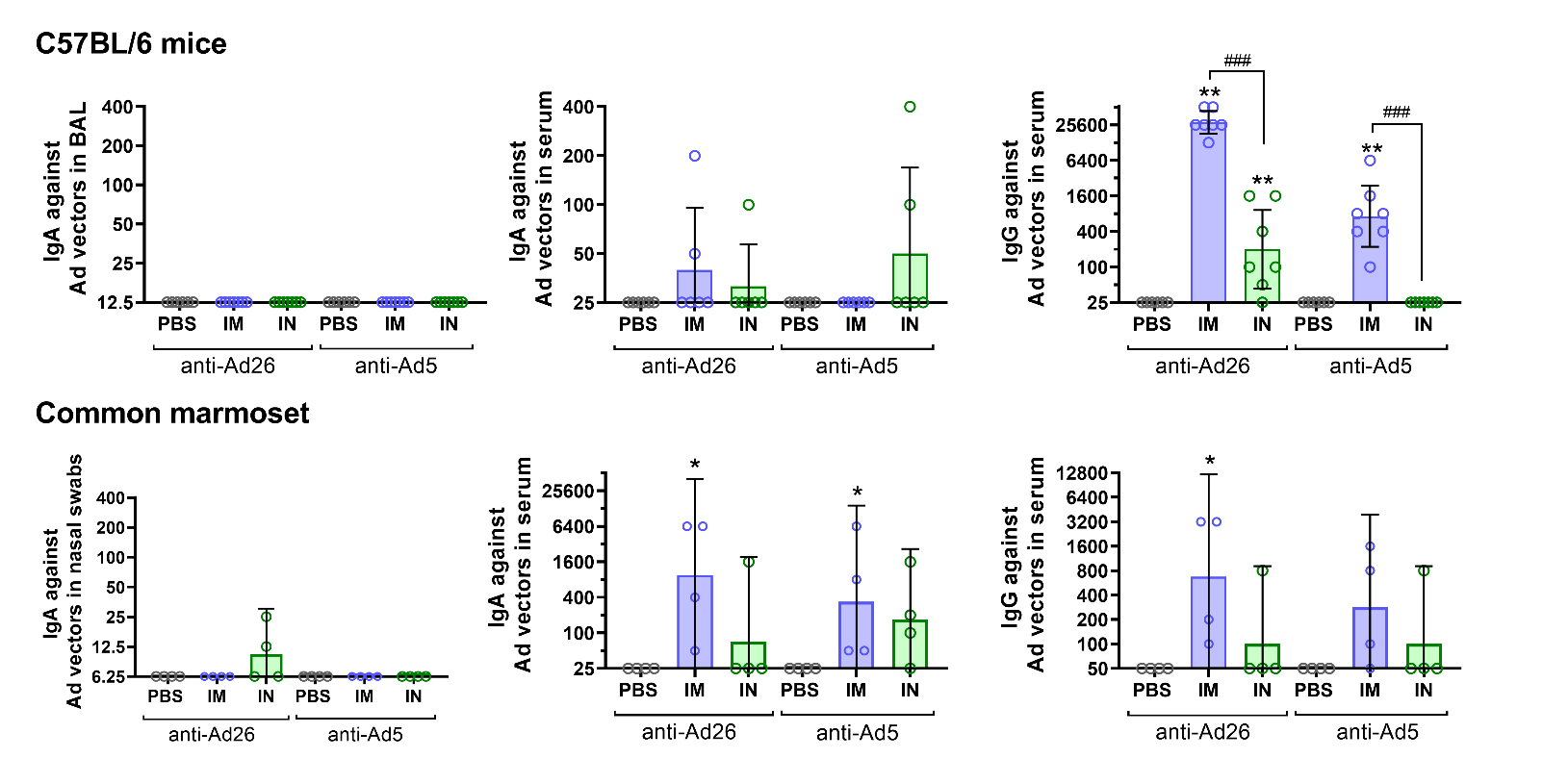


**Table S7 – Animal welfare scoring chart**

| Groups of parameters | Parameters assessed daily | Points | Comments |
| --- | --- | --- | --- |
| Location in the enclosure | Normal | 0 |  |
|  | Hiding in the upper corners | 1 |  |
|  | Does not leave the nest box | 3 |  |
| Activity | Normal | 0 |  |
|  | Increased activity | 1 |  |
|  | Increased aggression | 1 |  |
|  | Decreased activity | 1 |  |
|  | Ataxia, neurological symptoms (tremor, change in head position, stereotyped movements) | 5 |  |
|  | Unwillingness to move, cage support, difficulty accessing water and food, seizures | 10 |  |
|  | Position at the bottom of the cage / house with minimal response to stimuli, coma | 15 |  |
| Pose | Normal | 0 |  |
|  | Does not use one limb | 5 |  |
|  | Sitting with tail between legs | 2 |  |
|  | Limb injuries (depending on severity) | 1–10 |  |
| Feed and water consumption | Normal | 0 |  |
|  | Moderately reduced feed intake (no feed intake between feedings) | 1 |  |
|  | Markedly reduced feed intake (consumes less than half of the daily diet) | 3 |  |
|  | Complete loss of appetite | 5 |  |
|  | Moderate reduction in water consumption (less than 10 ml / day) | 1 |  |
|  | Complete refusal of water | 10 |  |
|  | Moderate increase in water consumption (more than 20 ml / day) | 1 |  |
|  | Marked increase in water consumption | 4 |  |
| Defecation and urination | Normal | 0 |  |
|  | Decreased amount of feces, dry feces, diarrhea, discolored feces, decreased urine volume and discoloration | 3 |  |
|  | Complete lack of feces | 8 |  |
|  | Anuria, blood in feces or urine | 10 |  |
| Skin and coat | Normal | 0 |  |
|  | Disheveled coat, hair loss, unkempt appearance | 3 |  |
|  | Rash, redness, yellowness, petechiae, abscesses, ulcers | 8 |  |
| Nose, mouth, eyes, head | Normal | 0 |  |
|  | Nasal discharge, hypersalivation, watery eyes, eye redness, ear inflammation | 3 |  |
|  | Loss of teeth, minor wounds of the oral cavity, adjusted dislocation of the mandible | 3 |  |
|  | Excessive salivation, salivation with traces of blood | 2 |  |
| Breathing | Normal | 0 |  |
|  | Rapid or slow breathing | 3 |  |
|  | Coughing, sneezing | 8 |  |
|  | Dyspnea, wheezing | 8 |  |
|  | Abnormal respiration with an open mouth, cyanosis, coughing up blood | 10 |  |
| Other changes | Specify: ______________________________________________ | 1–15 |  |

#### **Table S8 – List of antibodies against corresponding immunological markers used to assess the lymphocyte proliferative response by flow cytometry**

|  |  | **Fluorochrome** | **Clone** | **Manufacturer** |
| --- | --- | --- | --- | --- |
|  | Viability | DAPI | | Sigma Aldrich |
| Mouse | CD3 | PE-Cy7 | 145-2C11 | BD Biosciences |
|  | CD4 | PE | RM4-5 | BD Biosciences |
|  | CD8 | APC | 53-6.7 | BD Biosciences |
| Common marmoset | CD3 | AF700 | SP34-2 | BD Biosciences |
|  | CD4 | PE-CF594 | L200 | BD Biosciences |
|  | CD8 | PE | 6F10 | Biolegend |

#### **Table S9– List of antibodies against corresponding immunological markers to assess cytokine production in CD4 and CD8 cells by flow cytometry**

|  | **Fluorochrome** | **Clone** | **Manufacturer** |
| --- | --- | --- | --- |
| Viability | Zombie Violet™ Fixable Viability Dye | | Biolegend |
| CD45.2 | FITC | 104 | BD Biosciences |
| CD45 | BV786 | 30-F11 | BD Biosciences |
| CD8 | APC | 53-6.7 | BD Biosciences |
| CD4 | APC-Cy7 | GK1.5 | BD Biosciences |
| CD44 | PerCP-Cy5.5 | IM7 | BD Biosciences |
| IL-17 | BV605 | TC11-18H10 | BD Biosciences |
| IFNy | Pe-Cy7 | XMG1.2 | BD Biosciences |

**Histopathology assessment**

***1. ALI scoring system***

Lung tissue assessment was performed according to the ALI scoring proposed by the American Thoracic Society with slight modifications [4]. In particular, the changes included the elimination of one of the five assessment criteria, hyaline membranes, due to their almost complete absence in mice in lung injury models. In addition, we analyzed sections at 63x magnification, therefore, we changed the parameters to assess the presence of neutrophils in the alveolar and interstitial spaces. Below is a table that demonstrates the evaluation criteria for the ALI measurement system. The scores obtained from it were used to calculate the final ALI value using the formula: score = [(20 × A) + (14 × B) + (7 × C) + (2 × D)] / 86. The final score takes a value from 0 to 1, where 0 - is the absence of pathological changes, and 1 - is the maximum intensity of lung injury. We evaluated 10 sections for each mouse and summed the scores for all mice in the same experimental group. That is, the final sample for one experimental group is n = 30.

| Parameter | | Score | | |
| --- | --- | --- | --- | --- |
|  |  | 0 | 1 | 2 |
| A | Neutrophils in the alveolar space | - | 1 - 5 | >5 |
| B | Neutrophils in the interstitial space | - | 1 - 5 | >5 |
| C | Proteinaceous debris filling the airspaces | - | 1 | >1 |
| D | Alveolar septal thickening | <2x | 2х - 4х | >4x |

4. Gustavo Matute-Bello, Gregory Downey, Bethany B Moore, Steve D Groshong, Michael A Matthay, Arthur S Slutsky, Wolfgang M Kuebler, Acute Lung Injury in Animals Study Group (2011). An official American Thoracic Society workshop report: features and measurements of experimental acute lung injury in animals. Am J Respir Cell Mol Biol. 2011 May;44(5):725-38. doi: 10.1165/rcmb.2009-0210ST.

***2. Assessment of peribronchiolar and perivascular infiltration***

Another type of evaluation of pathological changes included the analysis of inflammatory infiltration. We analyzed the number of leukocytes in the peribronchiolar and perivascular spaces for 10 random bronchioles and 10 random vessels on a lung section of each mouse. The assessment was carried out according to the criteria indicated in table below. As a result, we received a value from 0 to 5, where 5 - is the maximum degree of tissue infiltration by leukocytes. The last step was, as in the previously described ALI criteria, the summation of 10 scores from 3 mice to obtain the final sample (n = 30).

| Score | The degree of manifestation of inflammation |
| --- | --- |
| 0 | Absolute absence of inflammatory infiltrate |
| 1 | The presence of single leukocytes |
| 2 | The presence of 1-2 groups of leukocytes |
| 3 | Infiltration of half of the bronchiole / vessel perimeter |
| 4 | Infiltration of most of the bronchiole / vessel perimeter |
| 5 | Infiltration of the entire perimeter of the bronchiole / vessel with radial strands of leukocytes |
